# Cation insertion to break the activity/stability relationship for highly active oxygen evolution reaction catalyst

**DOI:** 10.1038/s41467-020-15231-x

**Published:** 2020-03-13

**Authors:** Chunzhen Yang, Gwenaëlle Rousse, Katrine Louise Svane, Paul E. Pearce, Artem M. Abakumov, Michael Deschamps, Giannantonio Cibin, Alan V. Chadwick, Daniel Alves Dalla Corte, Heine Anton Hansen, Tejs Vegge, Jean-Marie Tarascon, Alexis Grimaud

**Affiliations:** 1 Chimie du Solide et de l’Energie, UMR 8260, Collège de France, 75231 Cedex 05 Paris, France; 2 State Key Laboratory of Optoelectronic Materials and Technologies, School of Materials, Sun Yat-Sen University, Guangzhou, 510275 People’s Republic of China; 3 Réseau sur le Stockage Electrochimique de l’Energie (RS2E), CNRS FR 3459,33 rue Saint Leu, 80039 Cedex Amiens, France; 4 Sorbonne Université, Paris, France; 5 Department of Energy Conversion and Storage, Technical University of Denmark, 2800 Kgs Lyngby, Denmark; 6 Skoltech Center for Electrochemical Energy Storage, Skolkovo Institute of Science and Technology, Moscow, 143026 Russia; 7 CNRS, CEMHTI UPR3079, Université d’Orléans, 1D avenue de la recherche scientifique, 45071 Cedex 2 Orléans, France; 8 Diamond Light Source, Harwell Science and Innovation Campus, Didcot, Oxfordshire OX11 0DE UK; 9 School of Physical Sciences, University of Kent, Canterbury, Kent CT2 7NH UK; 10 ALISTORE-European Research Institute, FR CNRS 3104, 80039 Amiens, France

**Keywords:** Electrocatalysis, Inorganic chemistry, Materials for energy and catalysis

## Abstract

The production of hydrogen at a large scale by the environmentally-friendly electrolysis process is currently hampered by the slow kinetics of the oxygen evolution reaction (OER). We report a solid electrocatalyst α-Li_2_IrO_3_ which upon oxidation/delithiation chemically reacts with water to form a hydrated birnessite phase, the OER activity of which is five times greater than its non-reacted counterpart. This reaction enlists a bulk redox process during which hydrated potassium ions from the alkaline electrolyte are inserted into the structure while water is oxidized and oxygen evolved. This singular charge balance process for which the electrocatalyst is solid but the reaction is homogeneous in nature allows stabilizing the surface of the catalyst while ensuring stable OER performances, thus breaking the activity/stability tradeoff normally encountered for OER catalysts.

## Abstract (web summary)

Renewable hydrogen production from water will require understanding and improving the oxygen evolution reaction (OER) on catalyst surfaces. Here, authors report α-Li_2_IrO_3_ to transform into a hydrated birnessite phase under OER conditions that exhibits enhanced OER performances and durabilities.

## Introduction

The spread of renewable energies has led to an ever-growing need to develop better electrochemical storage devices, such as electrolyzers^1–3^. This calls for key research advances to enhance the cost/performance ratio of electrolyzers, a cornerstone of which is improving the electrochemical reactions at the electrodes by the development of new catalysts. Nevertheless, several reports point toward the complexity of these reactions that involve the transfer of ions and electrons at the solid/liquid interface^4,5^. Considerable progress has thus been made toward designing efficient and noble metal-free hydrogen evolution reaction catalysts, but the other half reaction, namely the oxygen evolution reaction (OER), has proven to be more difficult^6–8^. Indeed, the OER is associated with large kinetics losses, where overpotentials >300 mV are typically obtained at a current density of 10 mA cm^−^²_oxide_ for the best-performing catalysts reported so far^6,9–11^.

Numerous research efforts were therefore devoted to improving the OER kinetics by developing transition metal oxides electrocatalysts. Recent experimental results and theoretical studies pointed out that increasing the transition metal–oxygen bond covalency leads to an enhanced OER kinetics concomitant with the destabilization of the surface^12–18^. Screening a large variety of transition metal oxides, it could be shown that this phenomenon is related to the relative position of the Fermi level when compared to the reversible potential for water oxidation, as well as to the charge-transfer energy^19–22^. In short, lowering the charge-transfer energy triggers the redox activity of oxygen ligand, leading to the involvement of lattice oxygen into the OER following the so-called lattice oxygen evolution mechanism. This involvement lowers the transition metal oxides coordination and thus destabilizes the surface of the catalyst, as now concluded by numerous research groups^23–27^. Furthermore, when the conduction band or the Fermi level of the transition metal oxides falls below the redox level of O_2_/H_2_O at the operating pH, a driving force is created for the catalyst to oxidize water^19,24^. Nevertheless, owing to the lack of charge balance mechanism, this reactivity is often counterbalanced by a dynamic cation dissolution/redeposition process and surface instabilities^5,28–30^. Hence, a tradeoff is observed where lowering the Fermi level of the catalyst below the reversible potential for water oxidation increases the catalyst reactivity with water at the expense of its long-term stability^13,26,31^. Eventually, a self-assembled surface composed of transition metal oxy(hydroxide) clusters is formed for these catalysts, as revealed by X-ray absorption spectroscopies (XASs)^26,30^ and high-resolution transmission electron microscopy (HRTEM)^32^, which evolves in a dynamic fashion upon OER conditions alike electrodeposited amorphous films^7,33–37^.

Strategies were thus recently developed in order to overcome this practical limitation associated with the activity/stability tradeoff for OER catalysts^38,39^. Among them, the design of nanostructured or supported catalysts appears promising to reduce the rate of dissolution^40–44^, with nevertheless the main limitation that the driving force for the transition metal oxide to be unstable is not suppressed.

In this work, we explore a chemical strategy that would enable breaking such activity/stability correlation. Herein, we develop an OER catalyst that Fermi level can be electrochemically lowered below the reversible water oxidation potential. Doing so, the catalyst chemically reacts with water at OER potentials, this reaction being counterbalanced by bulk cation exchange, thus preventing the structure decomposition/amorphization and ensuring a high stability while increasing the activity of the catalyst, when compared to the unreacted phase.

## Results

### Intercalation-stabilized catalysts for water oxidation

**Fig. 1:**
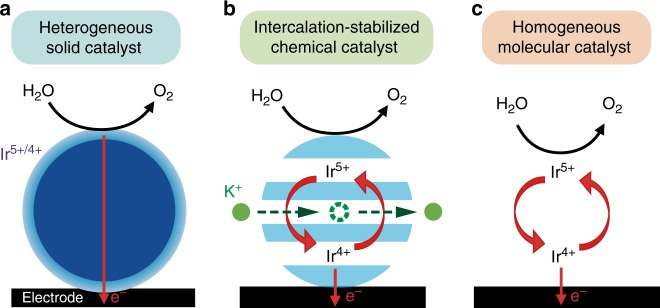
Charge compensated chemical oxidation of water. Schematic illustration of **a** heterogeneous OER process on the surface of solid catalysts, **b** the OER proceeding on the surface of a solid catalysts with the bulk of the catalyst being redox-active accepting cations from the electrolyte upon OER, and **c** homogeneous catalysis with soluble molecular catalyst.

**Fig. 2:**
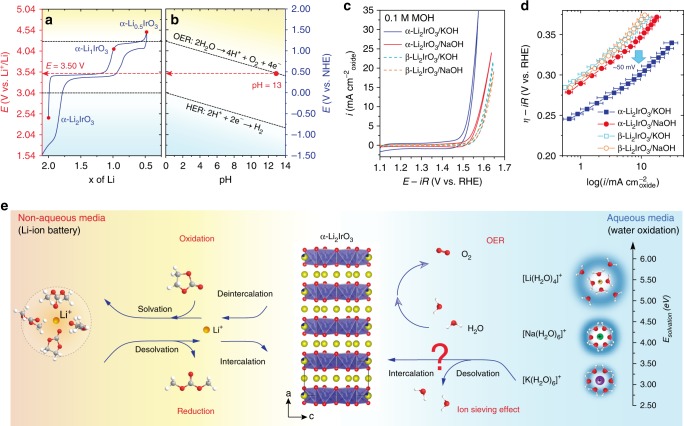
Electrochemical activity. **a** Cycling curve of α-Li_2_IrO_3_ as Li-ion battery electrode in organic solvent compared to **b** the Pourbaix diagram for water oxidation and reduction redox potentials as a function of pH. **c** CV curves of the layered α-Li_2_IrO_3_ phase compared to the 3D β-Li_2_IrO_3_ polymorph in 0.1 M KOH and NaOH aqueous solutions and **d** comparison of the corresponding Tafel plots. **e** Links between non-aqueous media (battery system) and aqueous media (water system) when using α-Li_2_IrO_3_ and β-Li_2_IrO_3_ as cathode materials in Li-ion battery and catalysts in aqueous for water splitting. The OER process in aqueous system may involve the intercalation and deintercalation of alkaline cations (such as Li^+^, Na^+^, and K^+^) in/out of the layered structure, which may be strongly affected by the desolvation energy of water molecules from solvated alkaline cations.

This new solid catalyst for which the bulk participates to the stabilization of the catalyst can be viewed at the frontier between heterogeneous and homogeneous catalysts^10,41,45^, hence we define them as intercalation-stabilized chemical catalysts (Fig. 1). Toward achieving the goal of breaking the activity/stability tradeoff for OER catalysts, we selected a transition metal layered oxide having an open two-dimensional crystal structure, α-Li_2_IrO_3_, and first studied its electrochemical behavior. The layered α-Li_2_IrO_3_ compound shows, when cycled in organic solvent, two successive oxidation events into α-Li_1_IrO_3_ and α-Li_0.5_IrO_3_ at ~3.5 V and 4.1 V vs. Li^+^/Li, respectively (Fig. 2a). The redox potential for the formation of α-Li_1_IrO_3_ corresponds to the thermodynamic potential for water oxidation at pH ≈ 13 (Fig. 2b)^46^, making it unstable toward water above this pH value. When using α-Li_2_IrO_3_ as OER catalyst in alkaline solution at pH 13, an irreversible oxidation peak is observed during the first cycle at ~1.23 V vs. RHE, corresponding to the formation of α-Li_1_IrO_3_ (Supplementary Figs. 1–3). Following this initial oxidation, a large OER activity with an overpotential as low as 290 mV at 10 mA cm^−^²_oxide_ is recorded in 0.1 M KOH (Fig. 2b, c). These numbers make α-Li_2_IrO_3_ one of the most active crystalline OER catalysts so far reported in alkaline conditions (Supplementary Fig. 4), validating a posteriori the choice of α-Li_2_IrO_3_.

### Structural and chemical analysis

Strikingly, the OER overpotential at 10 mA cm^−^²_oxide_ was found 50 mV higher as KOH is replaced by NaOH or LiOH (Fig. 2b, c, Supplementary Figs. 5 and 6). No such cation dependence is observed when changing the crystallographic structure of α-Li_2_IrO_3_ and using its 3D β-Li_2_IrO_3_ (ref. ^47^) polymorph (Fig. 2b, c, Supplementary Figs. 5 and 6) or for IrO_2_ (Supplementary Figs. 5 and 6). Accordingly, the Tafel slope was found lower for α-Li_2_IrO_3_ (≈50 mV/decade) in KOH than in NaOH, as well as lower than the ones measured for β-Li_2_IrO_3_ in both KOH and NaOH (all being ≈ 70 mV decade^−1^; Fig. 2c). This structural dependence for the observed cation dependence rules out an effect related to a shift of adsorption energies of OER intermediates, as previously reported^48–51^. Furthermore, no significant increase of the electrochemical active surface area or particle size is observed between NaOH and KOH (Supplementary Figs. 7 and 8), which excludes the effect of an increase of active sites to explain the enhanced OER performances, which rather originates from the in situ formation a new active phase at the OER potential, as discussed below.

**Fig. 3:**
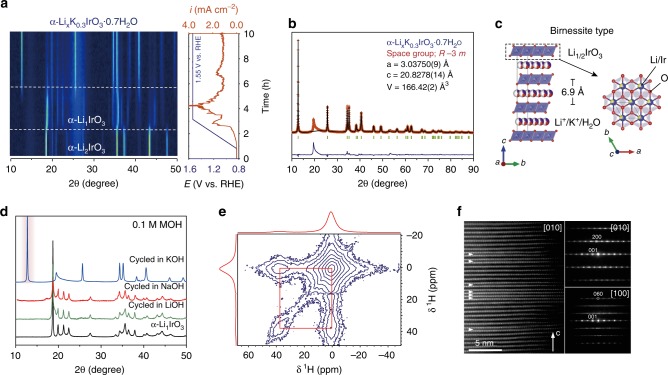
Characterization of the hydrated K^+^-containing birnessite phase formed during exposure with KOH. **a** Operando XRD performed during the electrochemical oxidation in 0.1 M KOH solution of α-Li_2_IrO_3_ from OCV (0.8 V) to 1.55 V vs. RHE at a scan rate of 0.1 mV s^−1^, and then hold for 10 h. **b** Rietveld analysis for the hydrated K^+^-containing birnessite phase, and **c** the corresponding structural model with K^+^ cations and H_2_O molecules distributed between the honeycomb Li_1/3_Ir_2/3_O_2_ layer. **d** XRD diffractograms collected for pristine α-Li_1_IrO_3_ and after cycling in different alkaline solutions (LiOH, NaOH, and KOH). **e** ^1^H 2D exchange spectroscopy (using a 10 ms mixing time) of the birnessite between interlayer H_2_O (0.9 ppm peak) and structural hydroxide groups (37.8 ppm peak). **f** Electron diffraction patterns (right) and HAADF-STEM image (left) of the birnessite phase. The O3 structure with d_001_ ≈ 6.2 Å interleaves with lamellas of the O1 structure with d_001_ ≈ 4.5 Å (marked with arrowheads).

Hence, to shed some light on both the effect of cation and the crystal structure, ex situ and in situ X-ray diffraction (XRD) studies were performed (Fig. 3a, Supplementary Fig. 9). In situ XRD first confirms the initial delithiation/oxidation and the formation of α-Li_1_IrO_3_ at ~1.23 V vs. RHE, followed by a shift of the first main 003 diffraction peak for α-Li_1_IrO_3_ at 2*θ* = 18.7° to a lower angle of 2*θ* = 12.6° concomitant with the OER (Fig. 3a). Rietveld analysis on ex situ XRD indicates the formation of a phase resembling a hydrated birnessite, such as the previously described K_x_MnO_2_·nH_2_O (ref. ^52^), with lattice parameters *a* = 3.03750(9) Å and *c* = 20.8279(14) Å (Fig. 3b). Within this model, disordered LiIr_2_ layers are stacked along the [001] direction and separated by 6.9 Å (Fig. 3c). Potassium atoms, water molecules and the possibly remaining Li are sandwiched and statistically distributed between the LiIr_2_ layers^53^.

Intrigued by this structural transformation, we then electrochemically prepared α-Li_1_IrO_3_ in water-free organic electrolyte before to react it with KOH. Doing so, the formation of the new phase with an O3-type layered structure (space group *R-3m*) was observed once again (Supplementary Fig. 10), indicating a chemical reaction of α-Li_1_IrO_3_ with the alkaline solution. However, no such transformation was measured when cycling α-Li_2_IrO_3_ or soaking the electrochemically prepared α-Li_1_IrO_3_ in LiOH or NaOH solutions (Fig. 3d, Supplementary Figs. 11 and 12), despite the smaller ionic radius of Li^+^ and Na^+^ compared to K^+^ (0.76 Å, 1.02 Å, and 1.38 Å, respectively). Similarly, no structural modification was recorded when soaking the oxidized 3D β-Li_1_IrO_3_ polymorph into KOH solution (Supplementary Fig. 12). Overall, these observations confirm the importance of the interplay between the crystallographic structure (α-Li_1_IrO_3_) and the alkaline cation (K^+^) for triggering the phase transformation and to enhance the OER activity of the catalyst (Fig. 2e).

To access the chemical composition of the newly obtained phase, energy-dispersive X-ray spectroscopy (EDX), high-temperature XRD, thermogravimetric analysis (TGA), and solid-state nuclear magnetic resonance (ssNMR) measurements were carried out. Sample prepared in 1 M KOH was analyzed by EDX and nearly 0.3 potassium per iridium atom was found (Supplementary Fig. 13), in agreement with the Rietveld refinement of the synchrotron XRD (Supplementary Table 2). High-temperature XRD indicates that the potassium-containing phase gradually evolves from 60 °C to 120 °C, leading to an intermediate phase with an interlayer distance of 6.24 Å (Supplementary Fig. 14). Complementary TGA analysis indicates that this phase change is associated with the loss of ~0.7 molecules of water per formula unit (Supplementary Fig. 15), giving an overall chemical formula close to Li_x_K_0.3_IrO_3_·0.7H_2_O (with *x* ≤ 1). From the relatively low temperature at which water starts to be released (60 °C), we can assume the presence of water loosely bound to the lattice as further confirmed by ssNMR measurements (Fig. 3e, Supplementary Fig. 16). Indeed, two proton environments could be found at 1.9 ppm and 37.6 ppm, corresponding to structural water and hydroxide groups, respectively. These two groups are in close proximity and likely exchange within the structure, as seen by the presence of crossed peaks in the two-dimensional NMR exchange spectrum. Overall, these measurements indicate that the structural water molecules as well as potassium cations are highly mobile within the birnessite structure. Therefore, to no surprise, this birnessite phase is highly unstable with structural potassium and water being easily removed during washing and drying steps (Supplementary Fig. 17), making the exact chemical composition highly dependent on the experimental conditions (see discussion in the Supplementary Table 2).

The local arrangement for this newly formed birnessite phase was then studied by electron diffraction (ED), bearing in mind that the intermediate dehydrated phase with *d* ≈ 6.2 Å previously spotted by high-temperature XRD is readily formed in the vacuum of the microscope. TEM study revealed a monoclinic O3 structure with *a* ≈ 5.28 Å, *b* ≈ 9.15 Å, *c* ≈ 6.37 Å, *β* ≈ 98^o^ (space group *C*2/*m*) that is reminiscent of α-Li_2_IrO_3_ but with a strongly enlarged interlayer separation (≈6.2 Å compared to ≈4.82 Å in α-Li_2_IrO_3_; Fig. 3f). High-resolution high-angle annular dark-field scanning transmission electron microscopy (HAADF-STEM) images also visualize numerous stacking faults that could be interpreted as insertions of thin lamellas with O1-type stacking and an interlayer separation as short as ~4.5 Å. This O1-type phase is also found as separate crystals, as confirmed by ED and HAADF-STEM imaging (Supplementary Figs. 18–20). Compositional STEM-EDX mappings reveal that the potassium cations are concentrated in the birnessite O3-type domains, whereas the O1-type phase contains no K^+^ (Supplementary Fig. 18). Therefore, we conclude that the broad peak found in the ex situ XRD at 20° (Fig. 3b) is reminiscent of potassium-free O1-type domains formed upon washing of the birnessite phase, since this peak was not observed by in situ XRD during the electrochemical formation of the birnessite O3 phase (Fig. 3a, Supplementary Fig. 9).

### The chemical oxidation process

**Fig. 4:**
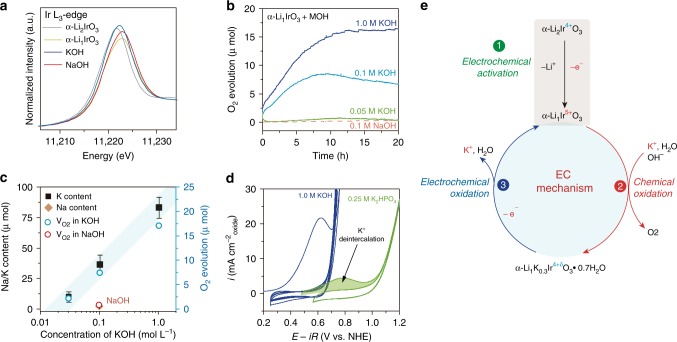
Chemical oxidation of water during birnessite formation. **a** Ir L_3_-edge XAS spectra probing the reduction of Ir after chemical reaction with KOH. **b** O_2_ gas evolution as detected by online mass spectrometry when soaking α-Li_1_IrO_3_ in KOH and NaOH solutions with increasing concentrations. **c** Linear relationship between the amount of intercalated K^+^ ions in the birnessite phase (as deduced by EDX) and the O_2_ gas evolution (as deduced from MS). **d** OER measurements in KOH at pH 14 compared to K_2_HPO_4_ at pH 9 demonstrating the K^+^ deintercalation from the birnessite phase at the OER potential. **e** Proposed EC mechanism for α-Li_2_IrO_3_, involving the electrochemical formation of a high valence α-Li_1_IrO_3_ oxide (*E* step), followed by the chemically oxidation of water to generate O_2(g)_ assisted by K^+^ intercalation into the birnessite structure (*C* step).

Having established that water and potassium are simultaneously inserted into the oxidized α-Li_1_IrO_3_ layered compound upon reaction with KOH, we then observe that the insertion is concomitant with a charge-transfer process with the alkaline electrolyte. To further elucidate this behavior, XAS measurements at the Ir L_3_-edge were first carried out (Fig. 4a). The shift of the white line observed for α-Li_1_IrO_3_ when compared to α-Li_2_IrO_3_ is consistent with the oxidation of Ir^4+^ to an oxidation state close to Ir^5+^ during the initial delithiation step, as previously observed^54,55^. Upon exposure of α-Li_1_IrO_3_ to 1 M KOH, the insertion of potassium into the iridium oxide and the formation of the birnessite phase is accompanied by the reduction of Ir back to an oxidation state comprised between 5 + (α-Li_1_IrO_3_) and 4 + (α-Li_2_IrO_3_). Hence, upon reaction with water, the catalyst is reduced, this process being selective to the cation since no reduction of Ir (i.e., no shift of the white line) was detected by XAS after soaking α-Li_1_IrO_3_ in NaOH (Fig. 4a).

Using mass spectrometry (MS), we unravel the chemical origin of such phenomenon. When in contact with KOH solution, gaseous oxygen is spontaneously generated from the chemical reaction of α-Li_1_IrO_3_, i.e., the oxidizing agent, with OH^−^ that acts as a reductant (Fig. 4b, Supplementary Fig. 21). We further observe that increasing the concentration of KOH has two effects. First, it increases the kinetics for oxygen release as seen by the increase of the initial slope for the oxygen evolution as measured by MS. Secondly, and more importantly, the quantity of gaseous oxygen generated by this chemical reaction is directly correlated to the KOH concentration. We also observe that the chemical reaction is stoichiometric, where the amount of oxygen detected by MS linearly correlates with the amount of K^+^ inserted into α-Li_1_IrO_3_ during its reduction at different concentration of KOH, with a ratio of approximatively 1:4, as expected for the four electron OER process (Fig. 4c). Furthermore, a large concentration of OH^−^ alone is not sufficient to chemically evolve gaseous oxygen, which also requires the use of adapted alkaline cations with small hydrodynamic radius and solvation energy (Fig. 2e), as well as open crystallographic structure, the combination of which being necessary to intercalate potassium and reduce α-Li_1_IrO_3_ (refs. ^56–58^). Hence, when using NaOH or the 3D β-Li_1_IrO_3_ polymorph, no oxygen is chemically generated from the reaction with water in alkaline conditions and no cation intercalation proceeds.

Finally, worth noticing that full conversion into the birnessite structure as well as chemical evolution of oxygen take time, as observed by in situ XRD and MS measurements (Figs. 3a and 4b, respectively). Looking into greater details by HRTEM at the K^+^ distribution on the surface of α-Li_2_IrO_3_, inhomogeneities are observed depending on the surface orientation and the availability of the diffusion channels (Supplementary Fig. 22), suggesting anisotropic diffusion properties of hydrated K^+^ cations inside the bulk structure.

While we demonstrated that the chemical reactivity of α-Li_1_IrO_3_ with KOH triggers the intercalation of hydrated K^+^, the question then arises regarding the reversibility of such process and if hydrated K^+^ is electrochemically deintercalated at the OER potential. For that, after an initial activation in KOH, OER measurements were carried out in K_2_HPO_4_ buffered solution at pH ≈ 9 (Fig. 4d). Doing so, an oxidation event can be observed at a potential corresponding to the OER onset potential in more strongly alkaline conditions. This observation is further confirmed by in situ XRD measurement that reveals the disappearance of the birnessite phase starting at ~0.6 V vs. NHE in K_2_HPO_4_ (Supplementary Fig. 23), while no such electrochemical oxidation was observed in Na^+^-containing electrolytes (Supplementary Fig. 24). Comparing the initial oxidation event measured in KOH and corresponding to the formation of α-Li_1_IrO_3_, the oxidation peak measured in K_2_HPO_4_ is limited, indicating that K^+^ is presumably only partially deintercalated and that the main active phase remains the birnessite one. Nevertheless, the oxidized α-Li_1_IrO_3_ form is partially regenerated during the OER. This regeneration will therefore trigger again the reactivity of the catalyst surface with water to generate gaseous oxygen and form again the birnessite phase. This observation indicates that, in addition to the classical electrochemical oxidation of water, this charge balance mechanism simultaneously occurs on the surface of the newly formed birnessite. However, this mechanism being associated with the transfer of 4 K^+^ and 4 e^−^ per mole of O_2(g)_ evolved, as we previously uncovered (Fig. 4c), the collection efficiency for oxygen evolution as measured by rotating ring disk electrode (RRDE) does not decrease when comparing measurements performed in NaOH and KOH (Supplementary Figs. 25 and 26), suggesting a stable catalytic process associated with oxygen evolution. This behavior is unlike the one previously observed for some other crystalline catalysts, such as perovskites and for which the collection efficiency was found to be degraded during surface reactivity^29,59,60^.

This charge balance mechanism can thus be schematically described as an electrochemical–chemical (EC) mechanism (Fig. 4e). During this EC mechanism, a high valence α-Li_1_IrO_3_ oxide is first electrochemically formed (*E* step) before to chemically reacts with water to generate O_2(g)_ (*C* step). It is worth stressing out that while the chemical oxidation of water on the surface of a high valence transition metal oxide is a thermodynamical event—the driving force for this reaction is given by difference between the redox potential for K^+^ insertion/deinsertion into the catalyst and the reversible potential for water oxidation—it is only made possible by counterbalancing the charge with the insertion of cations into α-Li_1_IrO_3_. To further understand the involvement of K^+^ into the electrochemical process, the effect of both K^+^ and OH^−^ concentrations on the overall OER kinetics was assessed independently. By keeping the pH equal to 13 and adding potassium salt to the solution, an increase of the OER activity is observed (Supplementary Fig. 27). Unlike for K^+^, when doing similar measurements with Na^+^ no enhancement of the OER activity was measured. From these measurements, we can conclude that the kinetics of the OER is dependent on the concentration of K^+^, which is needed to trigger the reformation of the birnessite phase that shows greater activity than the layered phase. Similarly, by mixing NaOH into 0.1 M KOH, hence keeping the concentration of K^+^ constant while increasing the concentration of OH^−^, the OER activity was found to increase (Supplementary Fig. 28). Looking into details at the Tafel plots for both sets of experiments, it can be seen that increasing the concentration of OH^−^ mostly affects the kinetics in the potential region limited by mass transport, while increasing the concentration of K^+^ affects both the mass transport limited region and the Tafel region. Nevertheless, the fact that the catalyst structure can reversibly switch from the layered to the birnessite structure, depending on the conditions renders the precise evaluation of the reaction orders for both K^+^ and OH^−^ uncertain.

### Computation study of OER intermediates

**Fig. 5:**
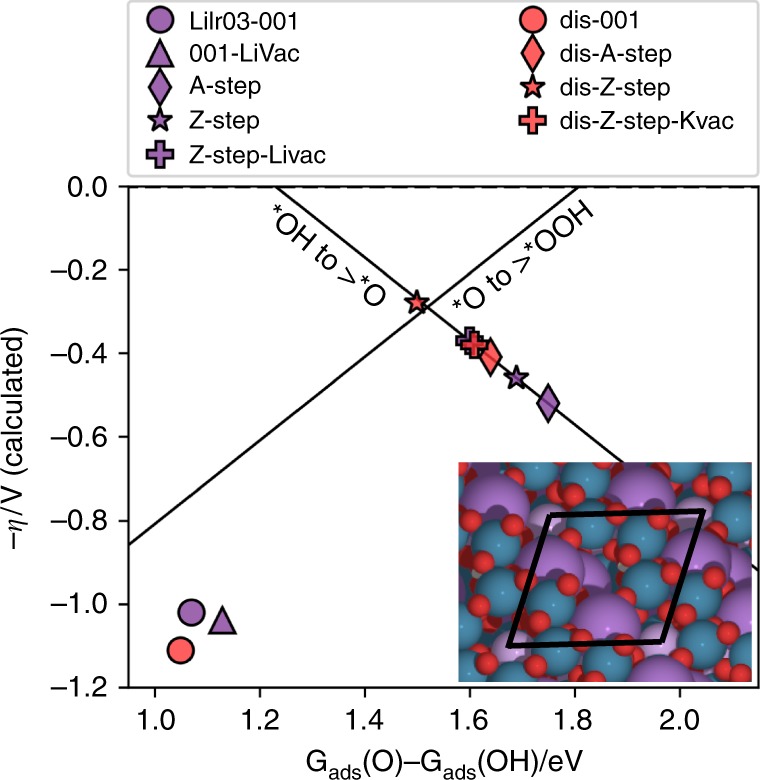
Calculated OER overpotential (*η*) as a function of *G*_ads_(O)–*G*_ads_(OH). Calculations for various surface sites on α-LiIrO_3_ (purple markers) and Li_0.75_K_0.25_(H_2_O)_0.50_IrO_3_ (red markers; see Supplementary Materials for further details on the structure of the various sites). Black lines indicate the volcano defined by the scaling relations between the adsorption energies of the ^∗^OH, ^∗^O, and ^∗^OOH intermediates. The inset shows the structure of the Z-step of the intercalated structure with the computational unit cell marked by black lines. (Ir is bluish gray, Li is pink, O is red, H is white, and K is purple).

Density functional theory (DFT) calculations were performed in order to assess the effect of this structural modification on the adsorption energies for the different OER intermediates *OH, *O, and *OOH on the surface of the iridate catalyst (see Supplementary Figs. 29–33 and Supplementary Tables 3–7 for further details). Since different surface terminations were observed experimentally by HRTEM, the adsorption energies as well as the OER overpotential (*η*) were calculated on the [001] surface of α-LiIrO_3_, as well as on two different kinds of steps with armchair and zigzag-type termination of the IrO_3_ layers (A-steps and Z-steps, respectively). The possibility of K, O, or Li vacancies was considered for all three surfaces, and the results for some plausible surface structures are plotted as purple markers in the volcano plot of Fig. 5 (see ESI for all the results). Points on the left leg of the volcano are limited by the formation of *OOH from *O, while points on the right leg are limited by the oxidation of *OH to *O. All sites on the [001] surface are found on the left leg of the volcano with rather large overpotentials, indicating that this surface is the less active for the OER. Instead, most of the step sites are found on the right leg or close to the top of the volcano. Corresponding models for the surfaces of the intercalated structure with stoichiometry Li_0.75_K_0.25_(H_2_O)_0.50_IrO_3_ were then made (see inset in Fig. 5) and the calculated overpotentials for these sites, plotted as red markers in Fig. 5, were found to be greatly modified by the intercalation of hydrated K^+^ into α-Li1IrO_3_. This finding indicates that the charge balance mechanism and the formation of the birnessite phase induces a change in the energies of the OER intermediates, and potentially in the OER kinetics with the calculated overpotentials being found on top of the volcano. However, comparing these calculated overpotentials with the ones computed for α-LiIrO_3_ on the same active sites, it remains complex to assign the enhanced OER activity to one specific active site.

### Electrochemical and structural stability

**Fig. 6:**
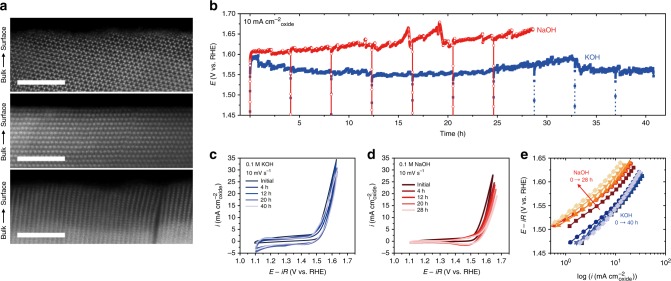
Stable surface and OER performances allowed by the potassium intercalation process. **a** Postmortem HRTEM images for α-Li_1_IrO_3_ continuously cycled 300 times in 0.1 M KOH at a scan rate of 10 mV s^−1^ within a potential range of 1.1–1.7 V vs. RHE. The scale bar is 1 nm. **b** OER performances with time for α-Li_1_IrO_3_ measured in 0.1 M KOH and compared with similar measurements performed in 0.1 NaOH. **c** and **d** show the CV curves of α-Li_1_IrO_3_ after certain times of OER performance in 0.1 M KOH and NaOH, respectively. **e** Corresponding Tafel curves.

Finally, we could conclude that, in addition to triggering the formation of the birnessite phase that shows greater OER performances, the charge balance process is essential for stabilizing this iridium-based catalyst and therefore break the activity/stability relationship. For that, HRTEM analysis was performed after 300 cycles in KOH and show that the surface of the catalyst remains perfectly crystalline with no formation of amorphous surface (Fig. 6a), unlike previous observations made for other highly active catalysts, such as Ba_0.5_Sr_0.5_Co_0.8_Fe_0.2_O_3−δ_ (refs. ^17,26,30,61^). Furthermore, negligible amount of iridium was found leached during the OER for α-Li_2_IrO_3_ (Supplementary Fig. 34). Therefore, the charge balance process allows for maintaining the structural integrity of the birnessite phase, which thus does not appear to show the obvious formation of self-assembled amorphous clusters on the surface of the catalyst. Hence, this stabilization process largely differs from the one previously observed for other transition metal oxides.

Furthermore, not only the catalyst’s surface is observed to be relatively stable, but the OER performances were found to be retained at a current density of 10 mA cm^−^²_oxide_ for over 40 h in 0.1 M KOH (Fig. 6b), unlike for NaOH for that it increases by 22% to 440 mV under similar conditions (Fig. 6b). This observation is reinforced by the analysis of cyclic voltammetry (CV) scans recorded every 4 h and that show no increase of the overpotential in KOH (Fig. 6c, e), unlike for NaOH for which the Tafel slopes show significant degradation of the OER performances for the layered phase (Fig. 6d, e, Supplementary Fig. 35). Hence, in addition to preserving the crystalline surface of the catalyst, the charge balance mechanism leads to sustained OER performances. In light of these results, mastering this charge balance phenomenon therefore provides a unique avenue for the future development of stable and active OER catalysts, which will call for a detailed investigation by integrating such catalyst in a practical AEMWE electrolyzer.

In summary, by a wise choice of open crystallographic structure combined with the use of alkaline cations with small hydrodynamic radius, we enable the electrolyte to be intercalated into the crystallographic structure of the layered iridium oxide during the chemical reactivity of the catalyst with water and therefore to participate to the overall charge balance phenomenon. Indeed, the use of in situ structural characterizations combined with gas analysis provide the demonstration that the chemical oxidation of water via the reactivity of the high valence oxide can be made reversible by the bulk insertion of hydrated K^+^ inside the α-Li_1_IrO_3_ structure, hence leading to the formation of a birnessite phase that possesses enhanced OER performances, while retaining perfectly stable structure and performances upon prolonged cycling. More importantly, similar bulk intercalation process governing the OER activity could be seen for another layered compound LiCoO_2_ (Supplementary Figs. 35–37), possibly making it a universal strategy to break the activity/stability relationship for OER catalysts. Hence, this work provides the fundamental understanding for experimental observations previously made for other layered compounds^62–65^ and helps to rationalize previous observations made about the effect of alkaline ions on the OER kinetics^48,49^. Finally, as usual with new findings, it asks for more theoretical development in order to fully uncover the potentiality of such mechanism in terms of performances, which will necessitate to include explicit solvent molecules as well as applied potential in order to fully capture this dynamic process.

## Methods

### Sample preparation

α-Li_2_IrO_3_ was synthetized by grinding IrO_2_ and Li_2_CO_3_ (in excess of 5%) according to literature^66^. To obtain α-Li_1_IrO_3_, α-Li_2_IrO_3_ is first charged to 4.0 V vs. Li^+^/Li at a constant current of 1/20 C in a Swagelok-type cell against metallic Li anode, using a glass fiber separator and LP100 electrolyte (1.0 M LiPF_6_ in ethylene carbonate (EC)/propylene carbonate (PC)/dimethyl carbonate (DMC) in a 1:1:3 weight ratio). The α-Li_1_IrO_3_ powder is then washed with DMC and dried at 25 °C. The synthesis of α-Li_0.5_IrO_3_ follows the same procedure, with the electrode (α-Li_2_IrO_3_) being charged to 4.6 V vs. Li^+^/Li.

The α-Li_x_K_0.3_IrO_3_·0.7H_2_O phase was synthesized by a chemical strategy. The above synthesized α-Li_1_IrO_3_ powder is soaked in a 1.0 M KOH solution for >12 h. The resulting powder is collected by centrifugation and first washed with ultrapure water and then with a water/acetone mixture (1:1 v/v ratio) before being dried at 55 °C overnight.

The birnessite α-Li_x_K_0.3_IrO_3_·0.7H_2_O sample was prepared by an in situ electrochemical method by cycling α-Li_2_IrO_3_ in KOH solution. Ir-For that, catalyst thin film with a mass ratio of 8:1:1 of α-Li_2_IrO_3_ powder to acetylene black carbon to Nafion(r) was drop-casted on a glassy carbon electrode (2 × 2 cm^2^). The electrode is cycled in 1.0 M KOH solution within a potential range of 1.1–1.7 V vs. RHE for OER at a scan rate of 10 mV s^−1^ for 50 cycles. After rinsing by excess amount of ultrapure water, the electrode is dried at room temperature. Powder is then collected from the glassy carbon electrode for further characterizations.

### Electrochemistry measurements

Electrodes were prepared by drop-casting an ink containing oxide catalyst powder on a glassy carbon electrode (5 mm diameter) or a large glassy carbon plate (2 × 2 cm^2^). The glassy carbon electrode was loaded with 0.5 mg_catalyst_ cm^−2^_disk_ and a mass ratio of 8:1:1 of Ir-oxide catalyst to acetylene black carbon to Nafion®. In general, 10 mg of Ir-catalyst powder was mixed with 1.2 mg of acetylene black carbon and ~60 μL of Nafion solution (5 wt.%) in 2 ml of tetrahydrofuran solution. Homogeneous catalyst ink was prepared by ultra-sonification for 10–20 min. A total of 10 μL of the as-prepared catalyst ink was then drop-casted on to a polished mirror-like glassy carbon electrode (5 mm in diameter), and dried in air at room temperature (25 °C).

OER measurements were carried out using a biologic MPG-30 potentiostat with rotating disk electrode (RDE, PINE Inc, US). The glassy carbon electrode loaded with catalyst is used as working electrode, and a KCl-saturated Ag/AgCl reference electrode (RE) and a Pt wire were employed as RE and counter electrode (CE), respectively. The Ag/AgCl RE is calibrated by using a reversible hydrogen RE (HydroFlex®, from Gaskatel, Germany) before and after each experiment. Potentials were then converted to the RHE scale. A scan rate of 10 mV s^−1^ was used for all CV and rotation was set to 1600 rpm. Oxygen was bubbled into the electrolyte before the CV and potentiostatic measurements. KOH solutions with different concentrations were prepared by Milli-Q® ultrapure water. Electrochemical measurements were conducted at room temperature (25 °C) otherwise mentioned. CV was also conducted at low temperature (4 °C) using a low-temperature thermostatic bath (Shanghai Selon Scientific Instrument Inc.) Tafel curves were plotted from OER kinetic currents, which were obtained from taking the average between forward and backward scans to remove the capacitive current contribution, and then corrected with ohmic losses. Ohmic drops were corrected by subtracting the ohmic voltage drop from the measured potential, using the electrolyte resistance determined by high-frequency AC impedance, where *iR*-corrected potentials are denoted as *E–iR* (*i* as the current and *R* as the electrolyte resistance). Error bars represent standard deviation from at least three independent measurements. Currents were normalized by the Brunauer, Emmet, and Teller (BET) surface area.

RRDE (PINE Inc., US) was employed to evaluate the efficiency of the OER. The applied potential on the Pt ring electrode is set to 0.4 V vs. RHE for detecting O_2_ generated at the disk electrode. Rotation was set at 1600 rpm. Solutions were saturated with argon before measurement. The collection efficiency is calculated as *η*=*i*_ring/_*i*_disc_ at a fixed potential (*E* = 1.58 V vs. RHE) where the oxygen evolution occurs.

Electrochemical stability of Ir-based catalysts was evaluated by both long-term CV cycles and chronopotentiometry. Chronopotentiometry was conducted at a current density of 10 mA cm^–2^_oxide_ in O_2_-saturated alkaline solutions for 40 h with CV being performed at different intervals.

In situ XRD were performed in a home^−^made electrochemical cell assembled on the platform of the BRUKER D8 Advance diffractometer. A three-electrode configuration is adopted using carbon paper loaded with ~5–8 mg cm^−2^_geo_ of α–Li_2_IrO_3_ catalyst as working electrode, a KCl-saturated Ag/AgCl RE and a Pt wire as CE. An X-ray transparent film of KAPTON^®^ 3511 (8-μm thick, from SPEX SamplePrep., US) is used to seal the electrochemical cell. After keeping the cell at open circuit voltage (OCV) for 40 min, the cell was charged to 1.55 V vs. RHE at a scan rate of 0.1 mV s^−1^, and then maintained at that potential for 10 h. Operando XRD measurements were carried out in the 2*θ* range of 10–50° during the above described electrochemical operations and patterns were collected every 10 min.

Li-ion batteries were assembled in an Ar-filled glovebox_._ The as-synthesized α–Li_2_IrO_3_ powder was used as active material, and mixed with 5% acetylene black carbon in a mortar. The mixture was dried in a Buchi oven at 80 °C under vacuum overnight prior to use. LP-100 (BASF) was used as electrolyte, in which 1.0 M LiPF_6_ is dissolved in EC/PC/DMC in a 1:1:3 weight ratio. Li-ion batteries were assembled using Swagelok-type cells with two glass fiber separators (Whatman, GF/D) and Li metal as CE. The assembled Li-ion cells were galvanostatically cycled using a VMP potentiostat (Biologic S.A., Claix, France).

### Characterizations

X-ray powder diffraction (XRD) measurements were performed using a BRUKER D8 Advance diffractometer in Bragg-Brentano geometry with Cu Kα radiation (*λ* Kα1 = 1.54056 Å, *λ* Kα2 = 1.54439 Å) and a LynxEye XE detector. All patterns were refined using the Rietveld method as implemented in the FullProf program. Evolution of XRD patterns with temperature were monitored in situ in the same diffractometer equipped with an Anton Paar HTK 1200N furnace to control the temperature. A ramping rate of 2 °C min^−1^ was applied and patterns collected every 20 °C, first, and then every 50 °C at higher temperature.

XAS measurements at Ir_III_ *L*-edge were carried out at B18 XAS beamline at the Diamond Light Source in the transmission mode.

ssNMR spectra were recorded on a Bruker 4.7 T Avance III spectrometer (i.e., 200 MHz for ^1^H) mounted with a 1.3 mm double-resonance probehead with Magic Angle Spinning at 60 kHz. The signal was detected with a rotor synchronized Hahn echo sequence over two rotor periods, with RF field strengths at 250 kHz. The chemical shifts were referenced with TMS at 0 ppm. The recovery delay was set to 1 s and 1024 transients were recorded. The longitudinal relaxation times T_1_ were recorded and found at 45 ms for the hydroxide peak ~37.6 ppm and 70 ms for the structural water peak ~1.9 ppm, ensuring that the measurements were quantitative.

TGA coupled with differential scanning calorimetry (TGA-MS) measurements were recorded with a STA 449C Netzsch apparatus under argon by applying a heating rate of 5 K min^−1^ from room temperature to 1000 °C using around 15–20 mg of material.

HAADF-STEM and EDX were performed on a FEI Titan 80 − 300 electron microscope operated at 300 kV and equipped with Super X detector. Samples for the TEM investigation were prepared by grinding powder samples in a mortar and dipping holey carbon TEM grids into the powder. ED patterns were acquired on a Philips CM 20 microscope operated at 200 kV. The morphology of the particles was observed by scanning electron microscopy performed in Hitachi S-3400N and FEI Quanta FEG 250 microscopes. EDX analysis was performed using an Oxford X-Max detector (accelerating voltage: 10 keV) to evaluate the atomic ratio of K to Ir. The specific surface area of each oxide was determined using BET analysis on a Quantachrome ChemBET Pulsar from a singlepoint BET analysis performed after 12 h outgassing at 150 °C.

Ir dissolution during OER was monitored using inductively coupled plasma (ICP) optical emission spectrometry on a ThermoFisher iCAP 6000 device. Chronoamperometry steps were applied holding the glassy carbon electrode loaded with 0.5 mg cm^−2^ catalyst at a constant voltage in 70 ml of 0.1 M KOH solution. The applied potential on the electrode was stepwise increased from 1.45 to 1.70 V vs. RHE, with each step (Δ*E* = 50 mV) holding for 20–40 min. After each chronoamperometry step, 1 ml of the electrolyte was taken out for the ICP measurement.

Online gas analysis was performed using a HIDEN H1 mass spectrometer system (HIDEN Analytical, UK). A three-neck Swagelok-type cell was used as the reaction container with one neck connecting to the mass spectrometer for gas analysis, two necks sealed by a stainless steel rod and a silicone rubber stopper. A total of ~50 mg of as-prepared α-Li_1_IrO_3_ powder was sealed in the Swagelok cell in the Ar-glovebox. The Swagelok cell was then connected to the HIDEN mass spectrometer through a flexible 1 m capillary inlet and two porous stainless steel membrane filters (~2 μm pore diameter, Valco Instruments Co. Inc.) for online gas analysis. A total of 2 ml of Ar-saturated KOH solution was injected using a syringe through the silicon stopper into the Swagelok cell. Blank test was conducted by injecting KOH solution into a sealed Swagelok cell without the α-Li_1_IrO_3_ powder.

### Density functional theory calculations

Spin-polarized DFT calculations were performed using the Vienna ab initio simulation package^67^, projector augmented-wave pseudopotentials and an energy cutoff of 585 eV. The atomic simulation environment^68^ was used to set up and analyze the structures. The exchange-correlation energy was described using the PBE functional^69^, which was found to reproduce the lattice constants of bulk α-Li_1_IrO_3_ well (c.f. Supplementary Table 3 in ESI). We furthermore find that the functional is able to reproduce the experimental layer separation of 6.94 Å in the disordered phase (Li_x_K_0.3_(H_2_O)_0.70_IrO_3_ (*x* ≤ 1) experimentally, calculated as Li_0.75_K_0.25_(H_2_O)_0.50_IrO_3_) reasonably well.

The brillouin zone of bulk α-Li_1_IrO_3_ is sampled using a 4 × 3 × 4 Monkhorst–Pack *k*-point grid while the *k*-point mesh for the surface models are chosen such that the products |a| *k_a_ and |b| *k_b_ are both >25 Å and a single *k*-point is used in the *c*-direction perpendicular to the surface. The unit cell dimension along the *c*-direction is chosen such that a minimum of 12 Å separates the adsorbates from the periodic image of the slab, and a dipole correction is used to decouple the electrostatic potentials across the periodic boundaries. All structures are relaxed until the forces on all atoms are below 0.02 eV Å^−1^.

Three different models of the surfaces are considered (c.f. Supplementary Figs. 29 and 30, Supplementary methods section in ESI); a 1 × 2 model of the [001] surface that corresponds to a termination that follows the layers, a [102] termination that results in an armchair-type edge of the hexagonal IrO_3_ layer (A-step), and a [111] termination that results in a zigzag-type edge of the IrO_3_ layer (Z-step). All surfaces are modeled by three layer slabs of the bulk structure, with the bottom layer fixed in the bulk position during optimization. Given the harsh oxidizing conditions, all Li is assumed to be stripped from the surface, and the slab is thus terminated by an IrO_3_ layer at the top.

The OER is assumed to follow the pathway:

(1) * + H_2_O → *OH + H^+^ + e^−^∆G_1_

(2) *OH → *O + H^+^ + e^−^∆G_2_

(3) *O + H_2_O → *OOH + H^+^ + e^−^∆G_3_

(4) *OOH → O_2_ + * + H^+^ + e^−^∆G_4_

where * denotes a surface active site, where the intermediate is bound to one or more Ir atoms. Since our starting point is the O-rich surface, at least one oxygen atom must be removed to form an active site (c.f. ESI for further details).

∆*G*_1_–∆*G*_4_ can be calculated from the adsorption free energy of the reaction intermediates, ∆*G*_ads_(*U*_vsRHE_), where the effect of potential, *U*_vsRHE_, is included through the computational hydrogen electrode (CHE):^70^

$$\Delta G_{{\mathrm{ads}}} = \Delta H_{{\mathrm{DFT}}}-T\Delta S + \Delta {\mathrm{ZPE}} + \Delta G_{{\mathrm{solv}}} - {\it{n}}eU_{{\it{{\mathrm{vsRHE}}}}}$$

Here ∆*H*_DFT_ is the change in enthalpy calculated from the DFT energies, ∆ZPE is the change in zero point energy, −*T*∆*S* is the energy contribution arising from the change in entropy, ∆*G*_solv_ is the change in solvation energy, and the last term is the CHE, with *n* being the number of electrons in the reaction and *e* being the numerical charge of an electron. From the adsorption energies, the limiting potential for OER can be calculated as:

$$U_{{\mathrm{lim}}} = {\mathrm{max}}\left\{ {\Delta G_1,\Delta G_2,\Delta G_3,\Delta G_4} \right\}/{\it{e}}$$

The ZPE and entropy terms, and the solvation energy have not been calculated explicitly, but an estimate has been included in the results presented in Fig. 5. The estimate is made from the ZPE and entropy terms calculated in ref. ^71^ (+0.35, +0.05, and +0.40 eV for the *OH, *O, and *OOH intermediates) and the solvation energies calculated for IrO_2_ in ref. ^72^ (ca. −0.10, 0.0, and −0.30 eV for *OH, *O, and *OOH) resulting in overall corrections of +0.25 eV, +0.05 eV, and +0.10 eV for *OH, *O, and *OOH, respectively. A comparison of the results with and without these corrections is given in the ESI. The energy of O_2_ in the gas phase is poorly described by GGA-type functionals, and is therefore corrected such that the free energy of formation of H_2_O is reproduced. Some of this error may also be present in the O–O bond of the *OOH intermediate, potentially giving rise to a small underestimation of the adsorption energy for this intermediate^73^.

## Supplementary information

Supplementary Information

## Data Availability

The data that support the findings of this study are available from the corresponding author upon reasonable request. DFT optimized structures can be obtained from 10.5281/zenodo.3632908.
