# Supplementary material for: Cation insertion to break the activity/stability relationship for highly active oxygen evolution reaction catalyst
Source: Nat Commun. 2020 Mar 13;11:1378. doi: 10.1038/s41467-020-15231-x (PMC7069983; doi:10.1038/s41467-020-15231-x)
Supplement: Supplementary file 1 — Supplementary Information [file 41467_2020_15231_MOESM1_ESM.pdf]

Supplementary Information

Cation Insertion to Break the Activity/Stability Relationship for Highly Active Oxygen Evolution Reaction Catalyst

Chunzhen Yang,[1,2] Gwenaëlle Rousse,[1,3,4] Katrine Louise Svane[5], Paul E. Pearce,[1,3] Artem M. Abakumov,[6] Michael Deschamps,[3,7] Giannantonio Cibin,[8] Alan V. Chadwick,[9,10] Daniel Alves Dalla Corte,[1,3,4] Heine Anton Hansen[5], Tejs Vegge[5], Jean-Marie Tarascon[1,3,4,10] and Alexis Grimaud[1,3]

Correspondence to: alexis.grimaud@college-de-france.fr

Supplementary Figures

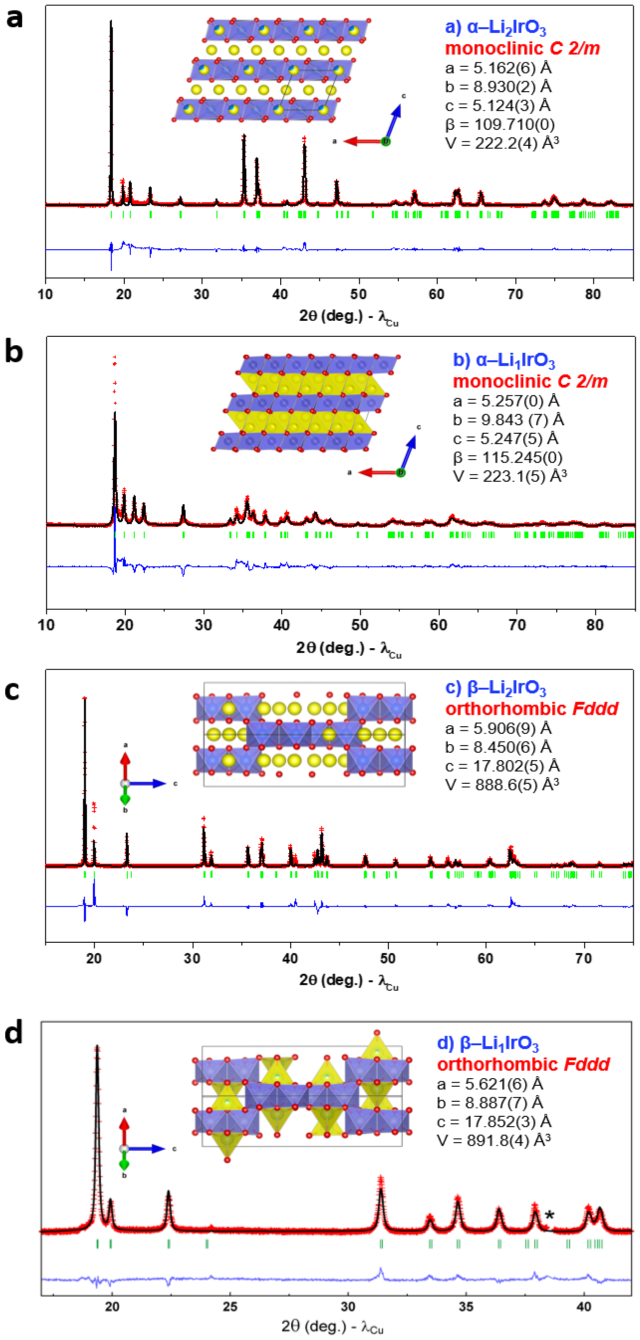

Supplementary Fig. 1. XRD patterns and Rietveld refinements for α-Li_2IrO_3, α-Li_1IrO_3, β-Li_2IrO_3, and β-Li_1IrO_3. The α-Li_2IrO_3 and its oxidized counterpart α-Li_1IrO_3 possess an expandable 2D layered structure, while both β-Li_2IrO_3 and β-Li_1IrO_3 have a relatively rigid 3D structure. This structural difference between these two polymorphs enables us to study the effect of the crystal structure on the ion-sieving effect for cation absorption/desorption during the dynamic OER process.

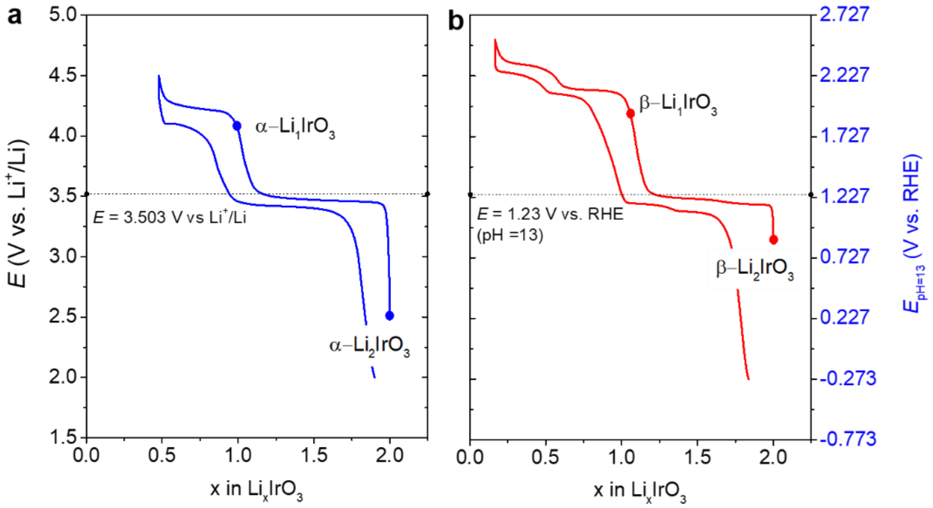

Supplementary Fig. 2. Galvanostatic charge-discharge voltage curves for (a) α-Li_2IrO_3 and (b) β-Li_2IrO_3 versus Li metal in Li-ion battery. Dashed line at E = 3.504 V vs. Li^+/Li indicates the OER reversible potential in aqueous alkaline solutions at pH = 13.

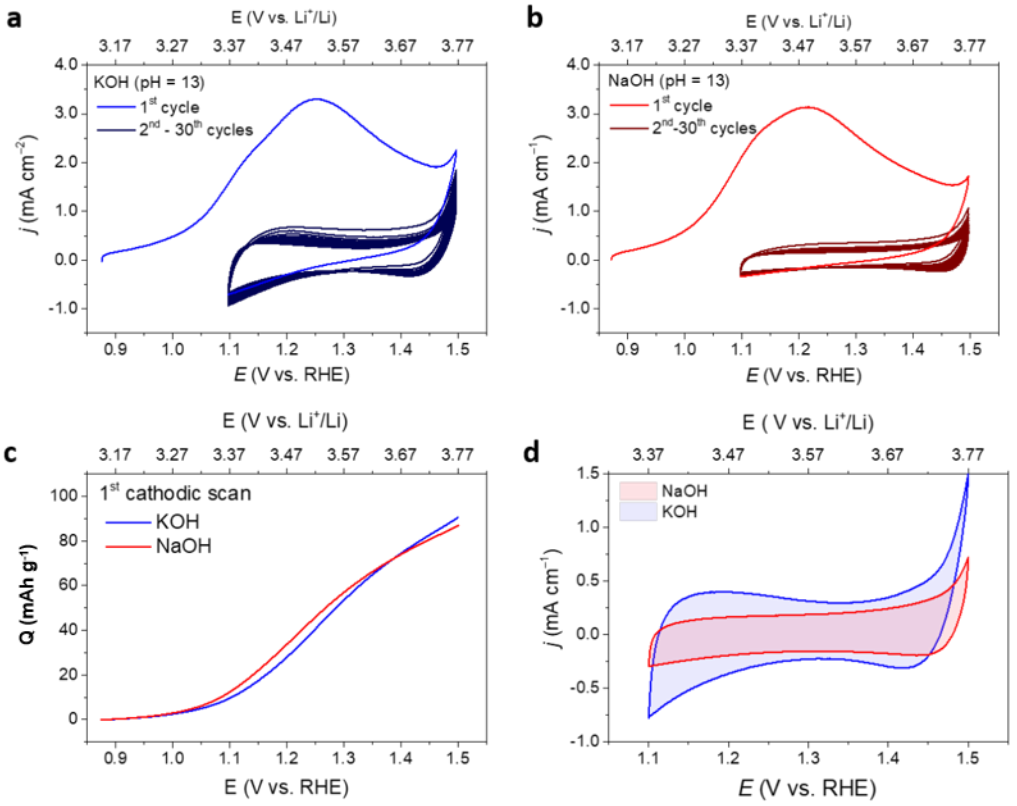

Supplementary Fig. 3. Electrochemical activation of α-Li_2IrO_3 in aqueous KOH and NaOH solutions (pH = 13). Cyclic voltammetry curves for α-Li_2IrO_3 in KOH (a) and NaOH (b) solutions. (c) Comparison of the charge capacitance for the 1st cathodic scan from OCV to 1.5 V vs. RHE in KOH and NaOH solutions. (d) Comparison of the double layer region between 1.1 – 1.5 V vs. RHE in KOH and NaOH solutions.

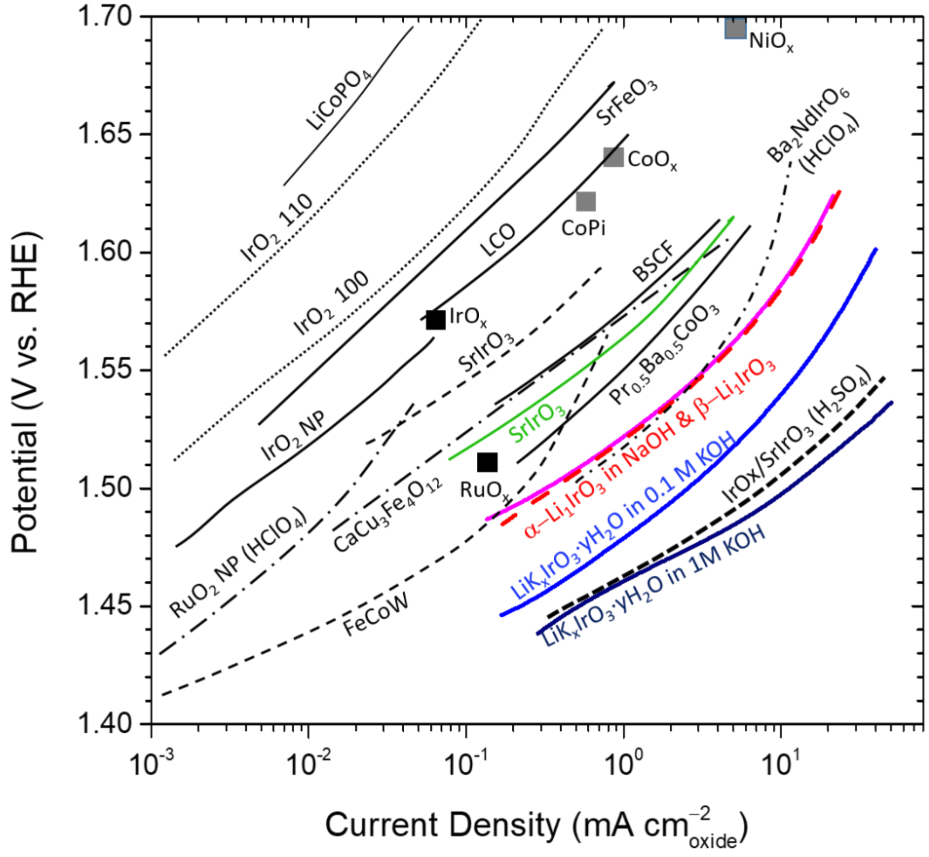

Supplementary Fig. 4. Tafel plot comparing the specific activities of different OER catalysts in alkaline electrolyte, as well as some of the best catalysts in acidic conditions. Colored lines are from this work. Gray and black lines/markers are duplicated from literature for $SrIrO_3$, $Pr_{0.5}Ba_{0.5}CoO_{3-\delta}$, $Ba_{0.5}Sr_{0.5}Co_{0.8}Fe_{0.2}O_{3-\delta}$ (BSCF), $CaCu_3Fe_4O_{12}$, IrO_2 nanoparticles, $LaCoO_3$ (LCO), $SrFeO_3$ and $LiCoPO_4$ in alkaline conditions (pH 13), as well as IrO_x/$SrIrO_3$ and Ba_2NdIrO_6 in acidic conditions[1-14].

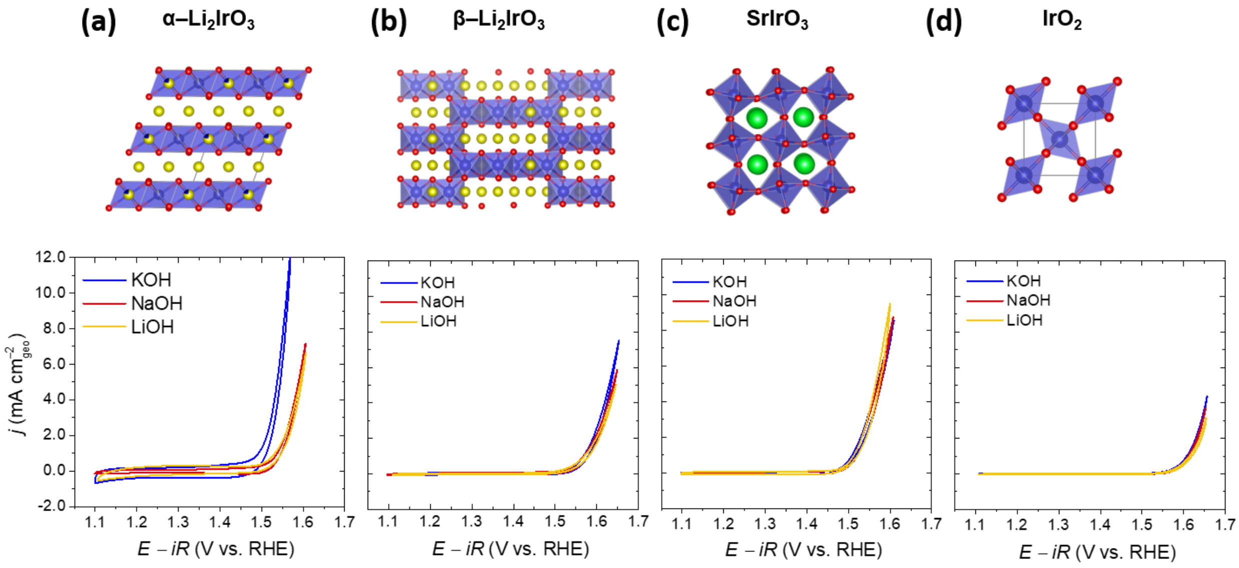

Supplementary Fig. 5. Cyclic voltammetry curves of various Ir-based catalysts, including (a) α–Li_2IrO_3, (b) β–Li_2IrO_3, (c) $SrIrO_3$ and (d) IrO_2, in 0.1 M of KOH, NaOH, and LiOH aqueous solutions. Among these model catalysts, the α–Li_2IrO_3 is the only one demonstrating an OER activity which is dependent on the cation from the supporting electrolyte.

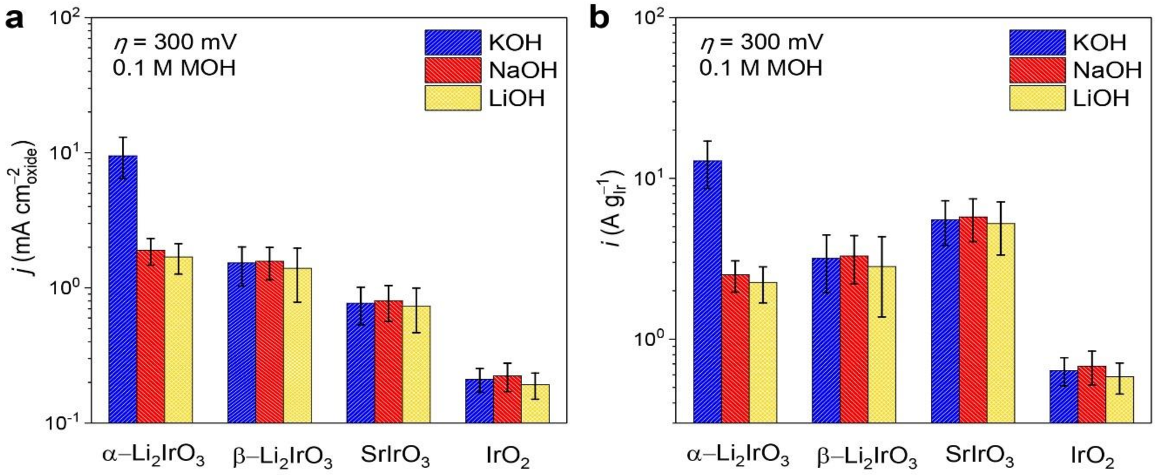

Supplementary Fig. 6. Comparison of the OER activities of different Ir-based catalysts in 0.1 M KOH, NaOH, and LiOH aqueous solutions. The OER currents at 350 mV overpotential are normalized by (a) the oxide surface area as determined by BET measurements and (b) the Ir amount.

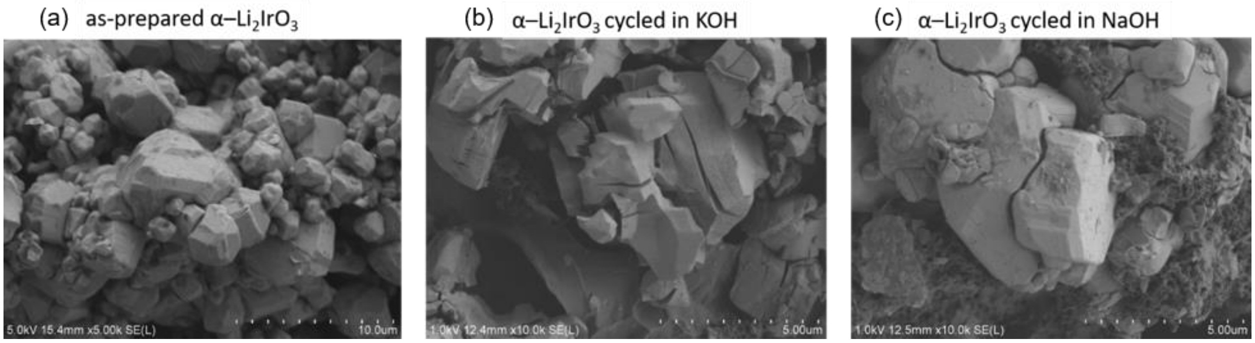

Supplementary Fig. 7. SEM images of pristine α–Li_2IrO_3 electrode (a) and cycled electrode in KOH (b) and NaOH (c) solutions.

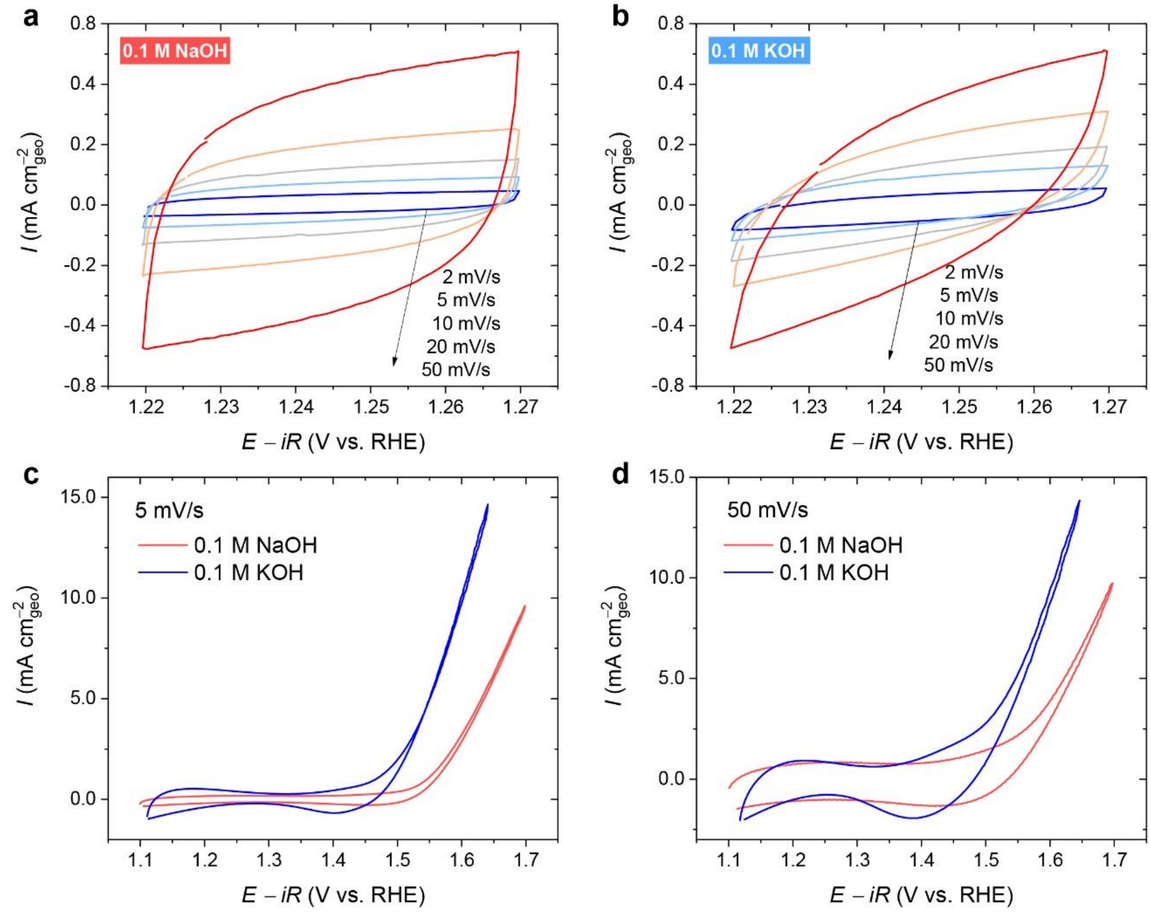

.

Supplementary Fig. 8. OER activity and ECSA estimation. (a-b) CVs measured for α-Li_2IrO_3 after a first activation step in NaOH followed by measurements in KOH in a limited voltage window between 1.22-1.27 V vs. RHE at varying scan rates. (c-d) Comparison of the OER activity in NaOH and KOH at 5 mV/s and 50 mV/s.

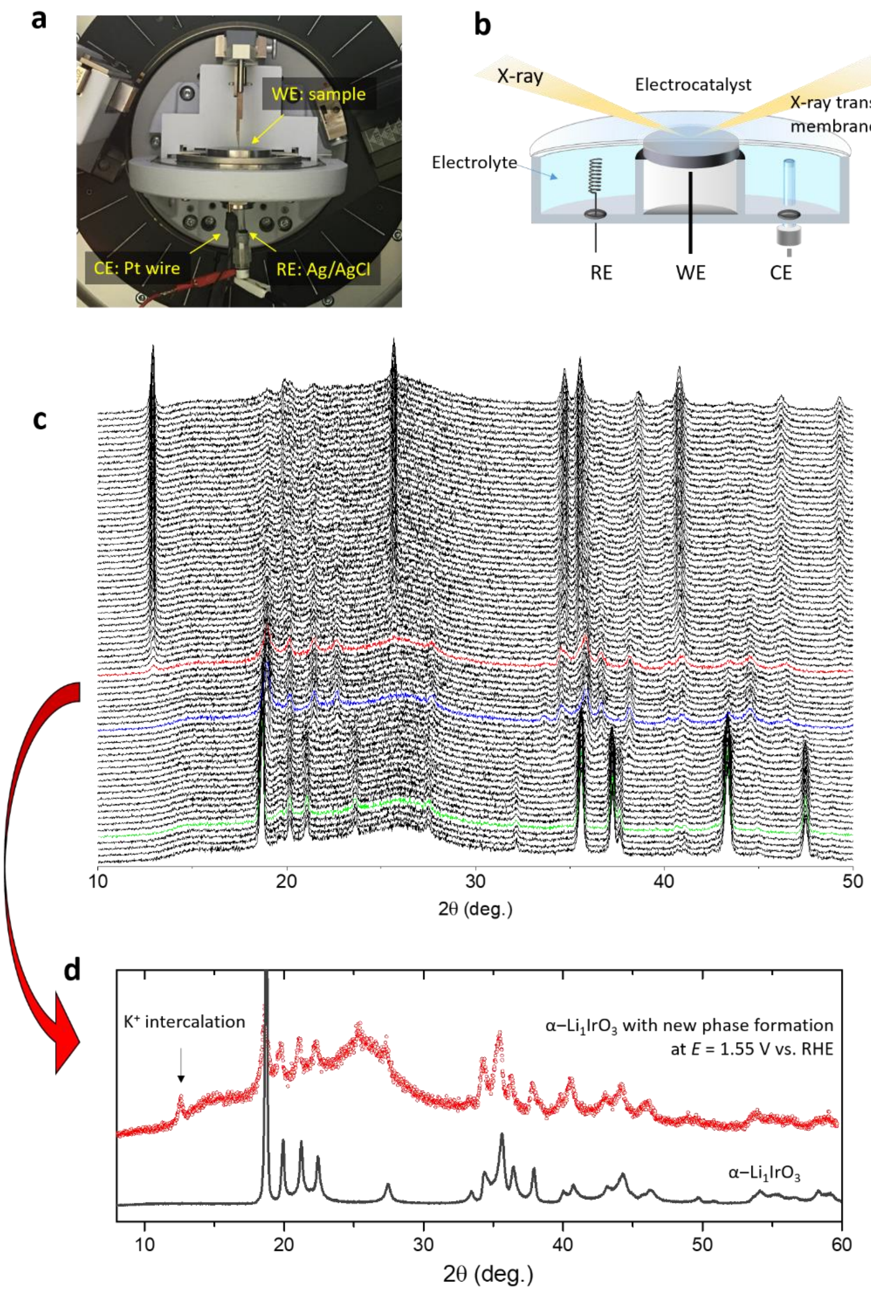

Supplementary Fig. 9. (a-b) *Operando* XRD setup and (c) XRD patterns for α–Li_2IrO_3 catalyst cycling in a 0.1 M KOH solution. (d) The corresponding XRD pattern labeled in red color.

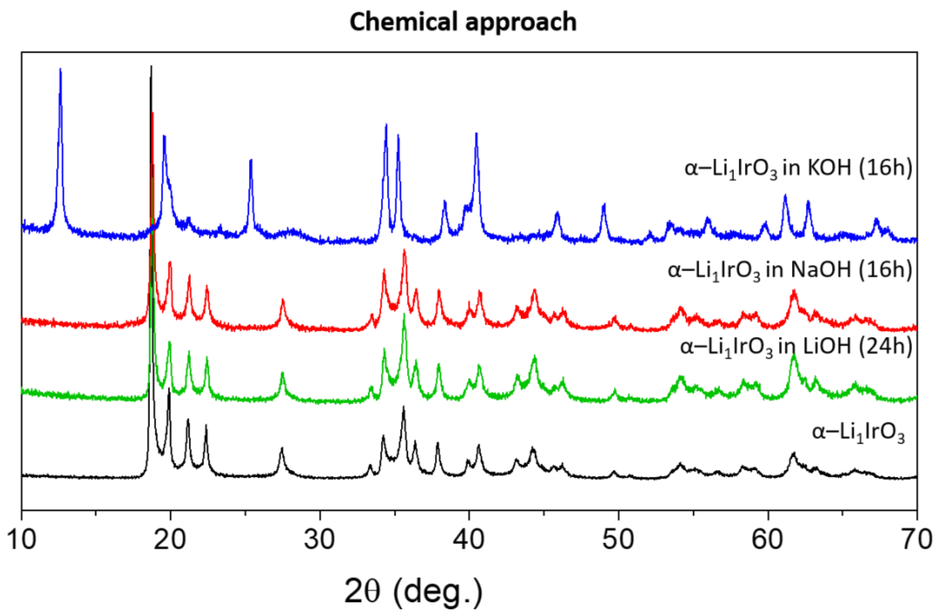

Supplementary Fig. 10. Chemical approach for the synthesis of the hydrated birnessite phase.

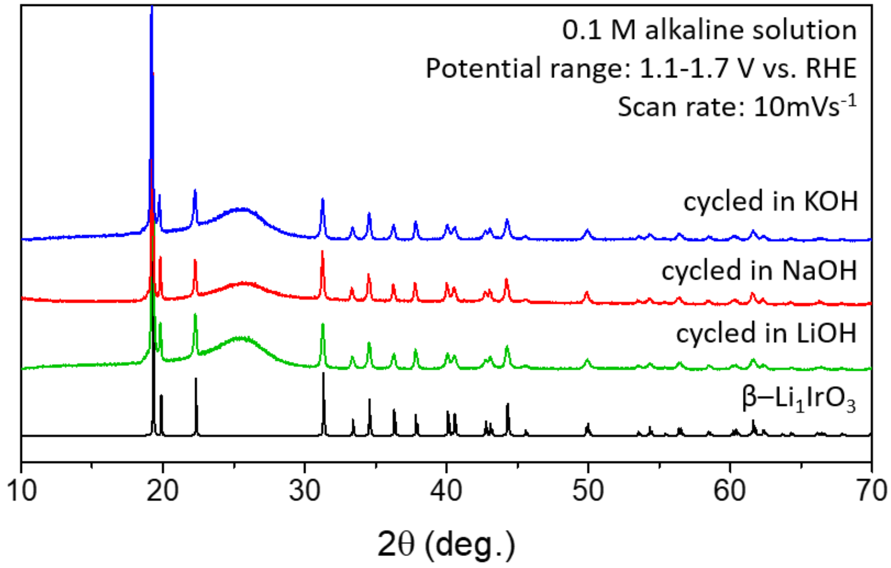

Supplementary Fig. 11. XRD for β-Li_2IrO_3 after cycling under OER conditions in 0.1 M KOH, NaOH, and LiOH. After 50 cycles at a potential range between 1.1 – 1.7 V vs. RHE, the β-Li_2IrO_3 catalyst transforms to β-Li_1IrO_3 but no further evolution to a new phase is recorded, unlike for α-Li_2IrO_3.

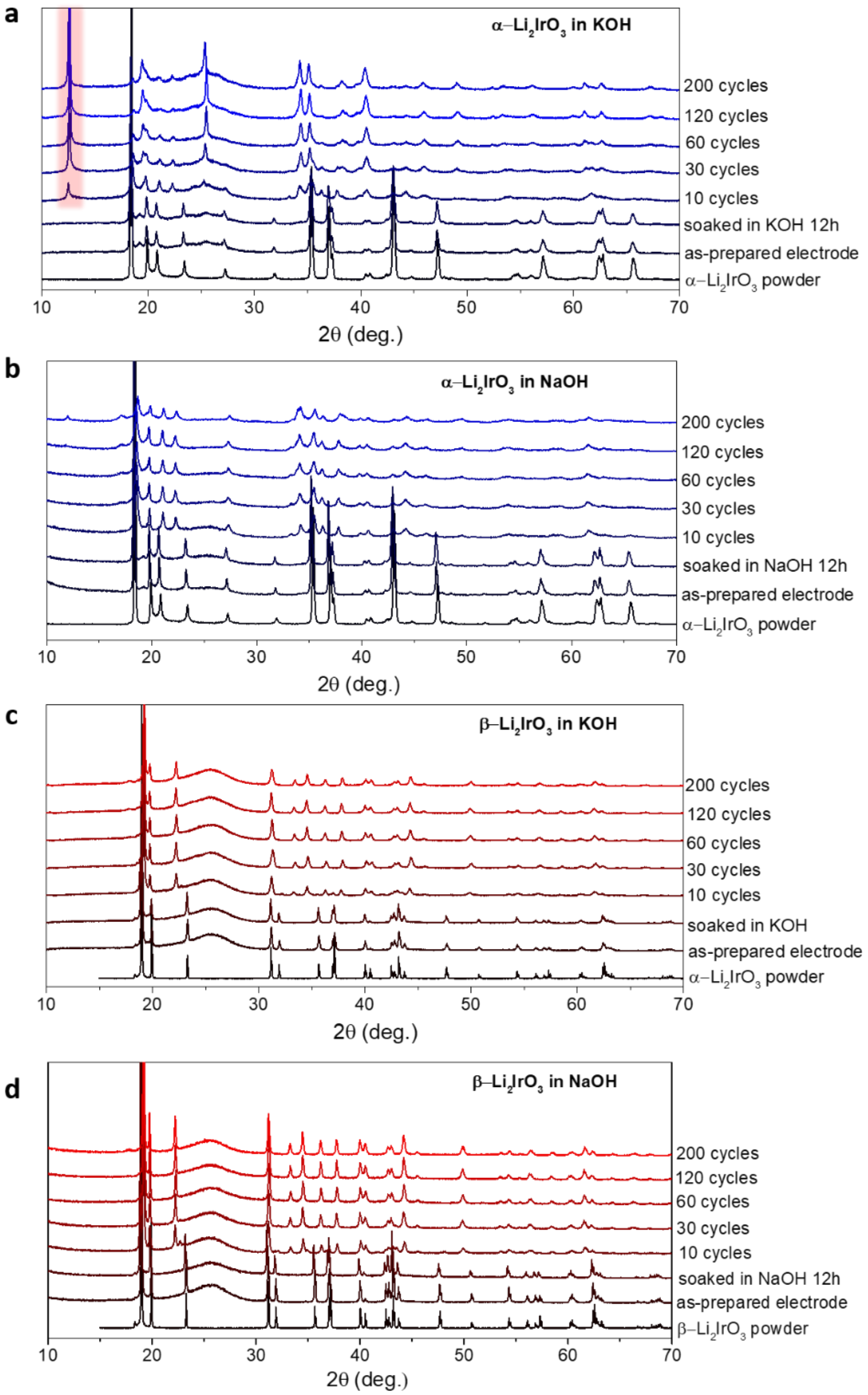

Supplementary Fig. 12. Structural evolutions recorded by *ex situ* XRD for α-Li_2IrO_3 and β-Li_2IrO_3 when used as OER electrocatalysts and cycled in 0.1 M KOH and NaOH solutions from OCV (soaked) up to 200 cycles.

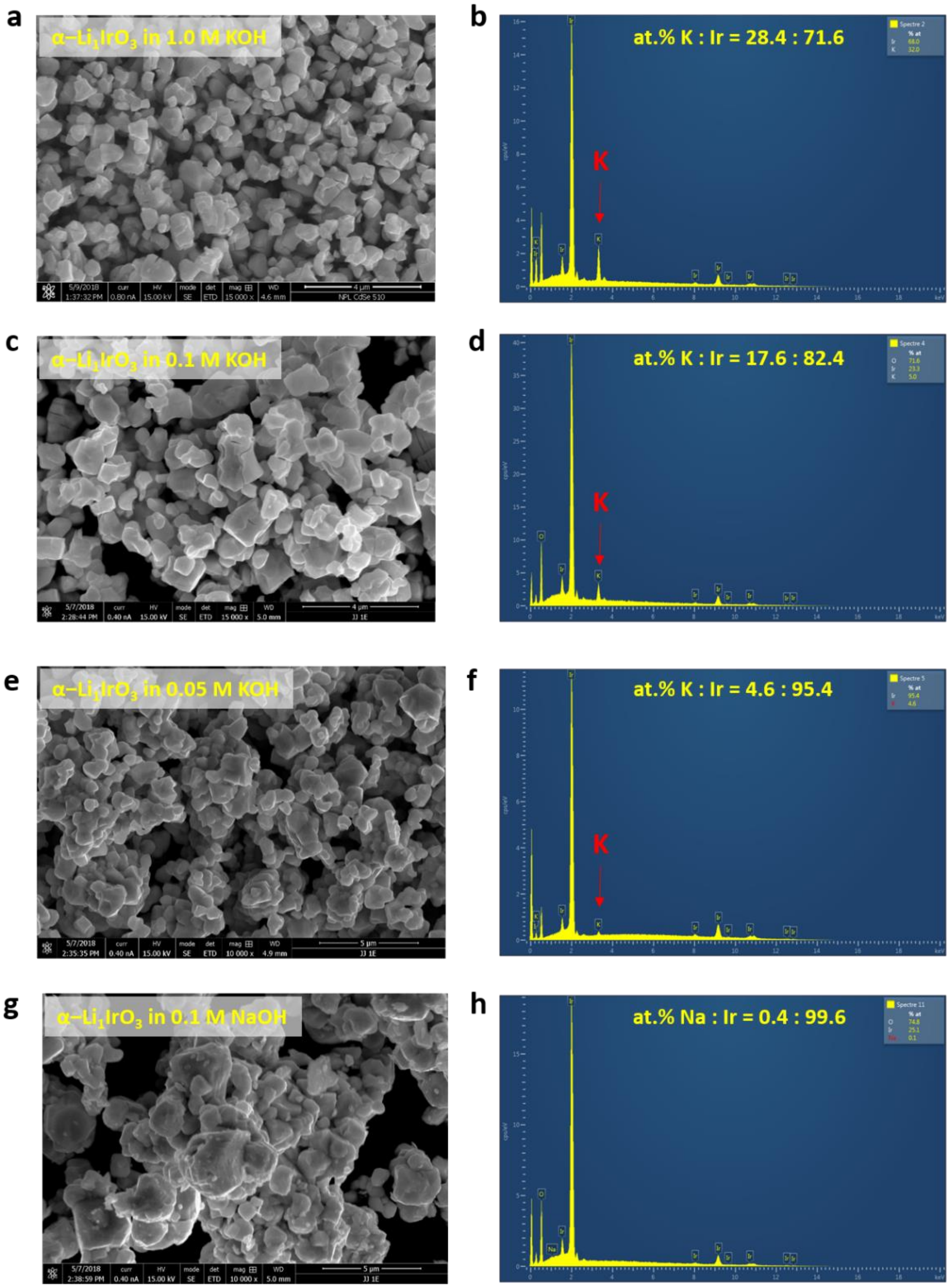

Supplementary Fig. 13. SEM images and EDX analyses for α-Li_1IrO_3 powder after being soaked in KOH (a-b) 1 M, (c-d) 0.1 M, (e-f) 0.05 M and NaOH 0.1 M (g-h) aqueous solutions.

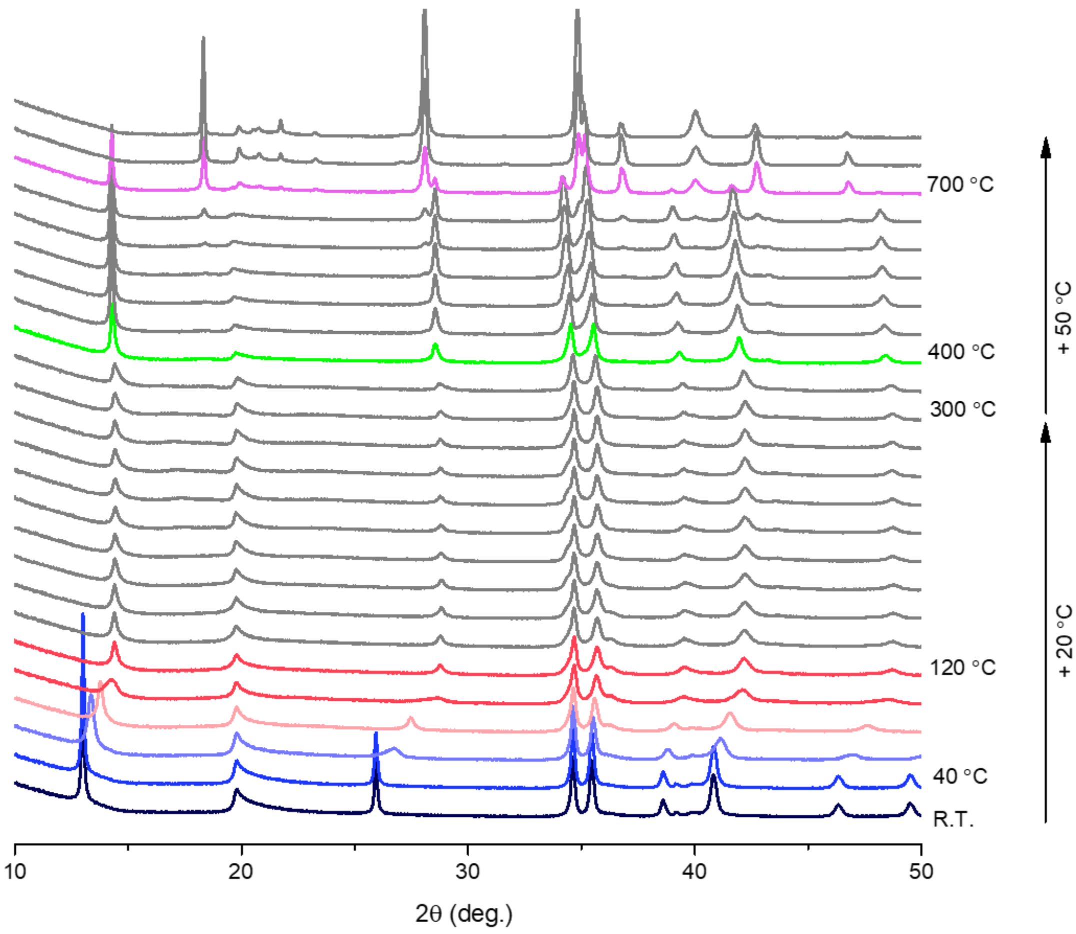

Supplementary Fig. 14. High Temperature XRD profiles showing the structural evolution of the hydrated birnessite phase with increasing temperatures. The shift of the 003 peak initially at 2θ = 12.6° (d = 6.9 Å) to 2θ = 14.4° (6.1 Å) is associated with a decrease of the interlayer distance and the loss of structural water to form a dehydrated K^+-containing intermediate.

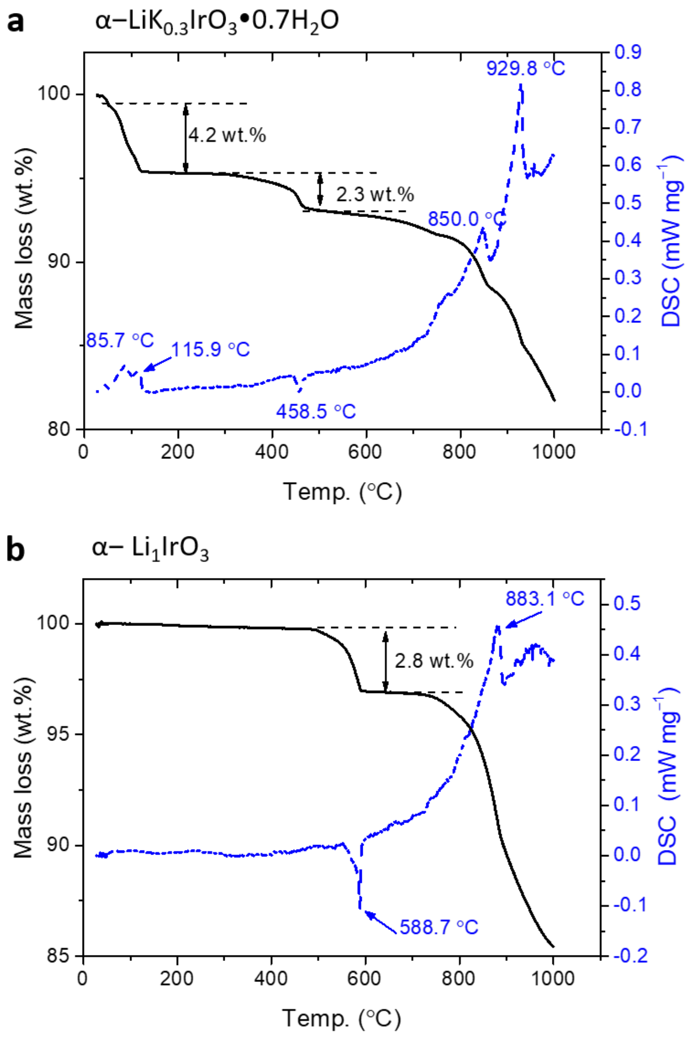

Supplementary Fig. 15. TGA analyses of (a) α-$LiK_{0.3}IrO_3 \cdot 0.7H_2O$ and (b) α-Li_1IrO_3.

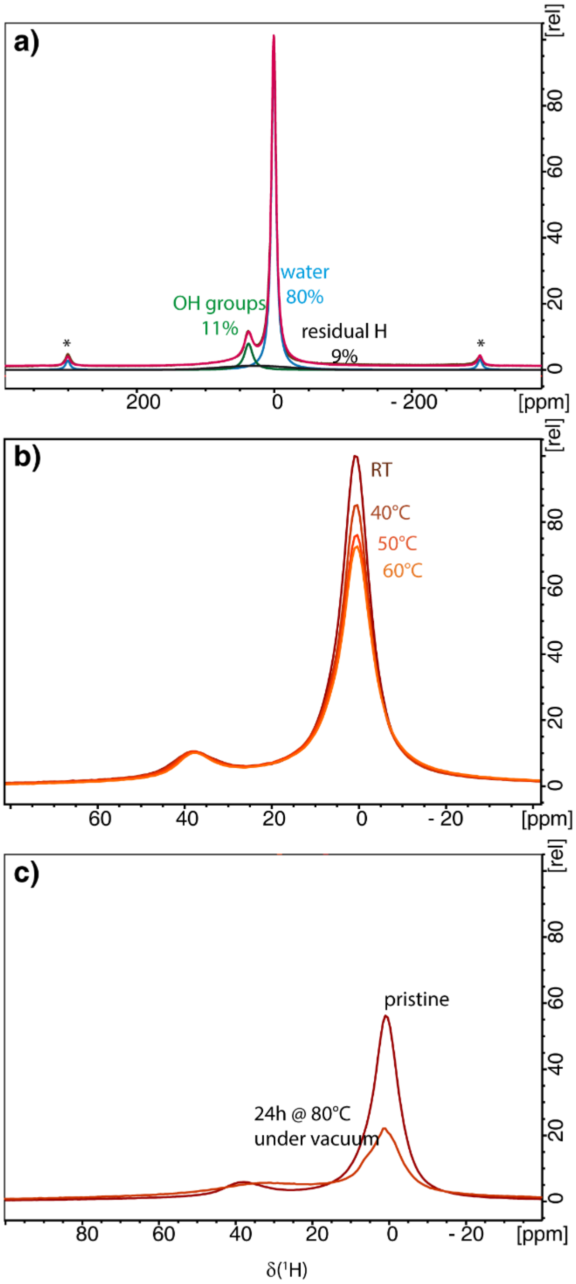

Supplementary Fig. 16. ^{1}H solid-state NMR (ssNMR) analysis of the hydrated birnessite phase. (a) deconvolution of the ^{1}H spectrum with three proton environments, with residual protons accounting for 9%, structural OH groups accounting for 11% and structural water for 80%. (b) Upon heating in the spectrometer, the intensity for structural water peak at 1.9 ppm slowly decreases indicating that structural water is loosely bond to the lattice. (c) Under vacuum, such as encountered in the microscope for TEM, the peak for structural water at 1.9 ppm keeps decreasing, indicating that the hydrated birnessite phase transforms into a dehydrated intermediate under vacuum.

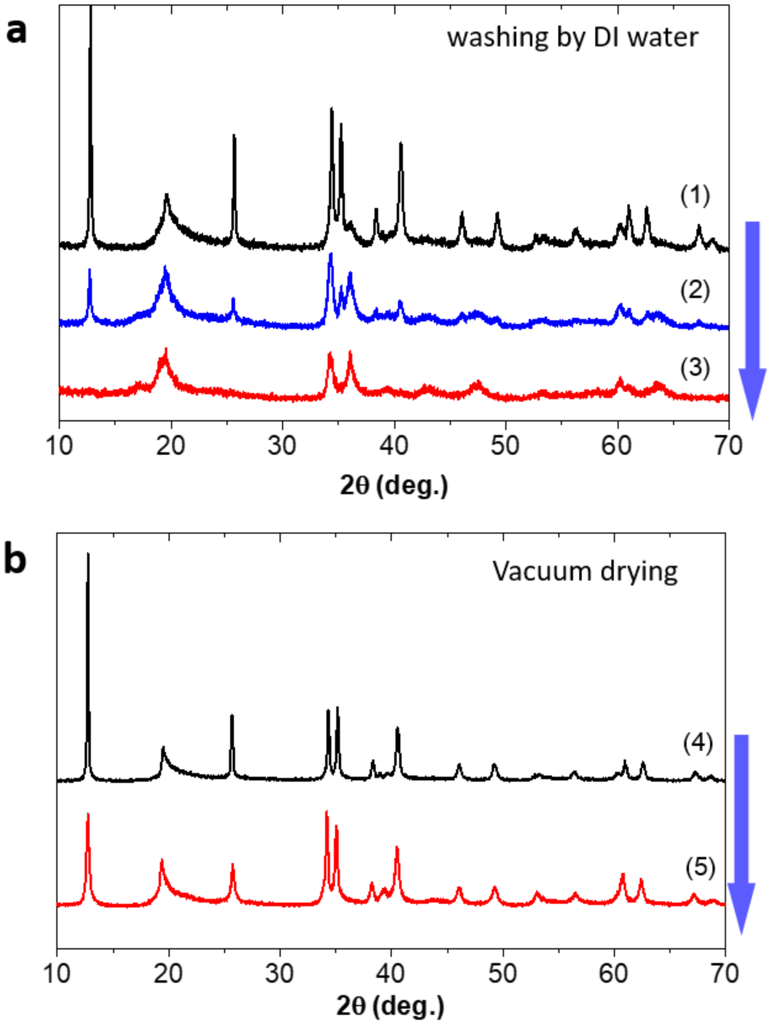

Supplementary Fig. 17. XRD patterns demonstrating the instability of the birnessite O3-type phase upon washing. XRD diffractograms showing the structural evolution of the hydrated birnessite phase after repeated washing by DI water (a) and vacuum drying (b). (1) as-prepared pristine birnessite sample washed by a mixed H_2O/acetone (1:1) solution, (2) washed with DI water for 3 to 5 times till the pH reaches ~9, (3) washed with DI water till the pH reaches neutral, (4) as-prepared pristine birnessite sample washed by mixed H_2O/acetone (1:1), and dried at R.T., (5) sample dried in a vacuum oven at a pressure of 50 mbar at 40 °C for 4 hours.

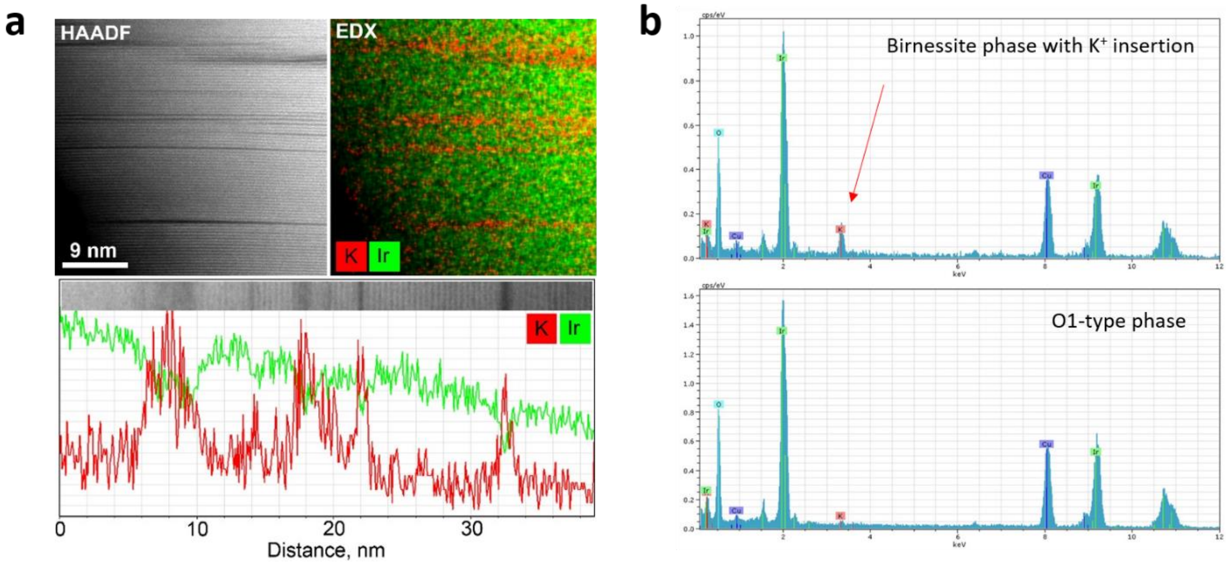

Supplementary Fig. 18. (a) EDX mapping of K and Ir elements for the birnessite $Li_xK_{0.3}IrO_3{\cdot}0.7H_2O$ showing a crystal with mostly the O1 domains as well as some O3 intergrowth. (b) Corresponding EDX spectra showing the presence of K^+ only in the O3 domains while the O1 domains contain no K^+. The Cu signal originates from the specimen support. From the EDX mapping it can be concluded that K^+ intercalation is observed for the expanded O3-type phase while the collapsed O1 domains formed during the washing steps contain no K^+.

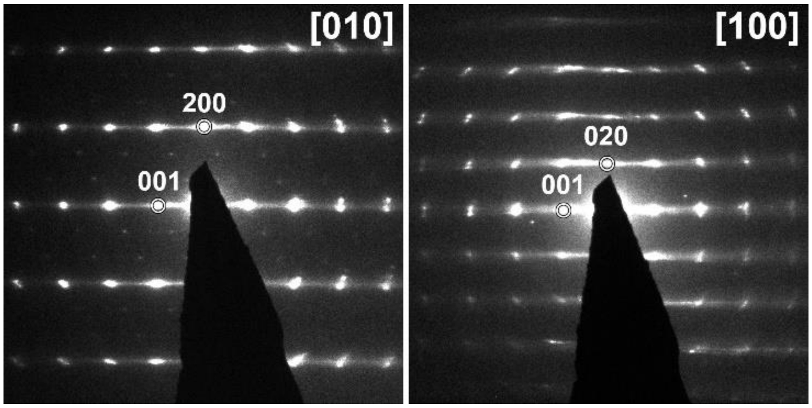

Supplementary Fig. 19. Electron diffraction patterns of the O1 phase indexed with a monoclinic C2/m unit cell with a ≈ 5.29Å, b ≈ 9.03Å, c ≈ 4.47Å, β ≈ 90º.

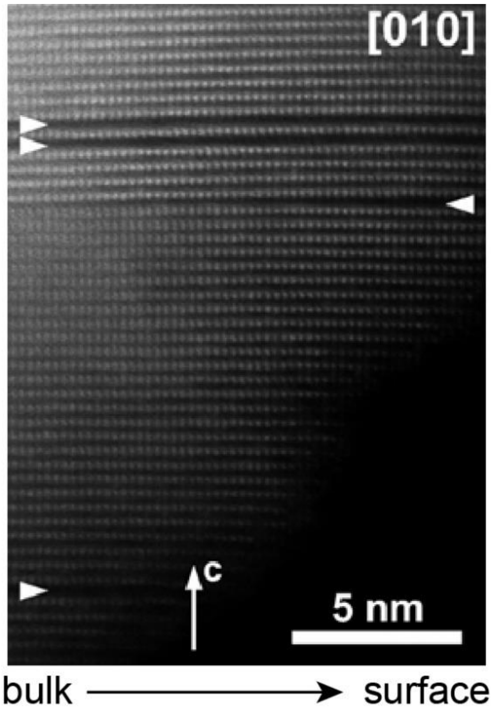

Supplementary Fig. 20. [010] HAADF-STEM image of the O1 phase. The occasionally occurring lamellas with enlarged interlayer separation are marked with arrowheads.

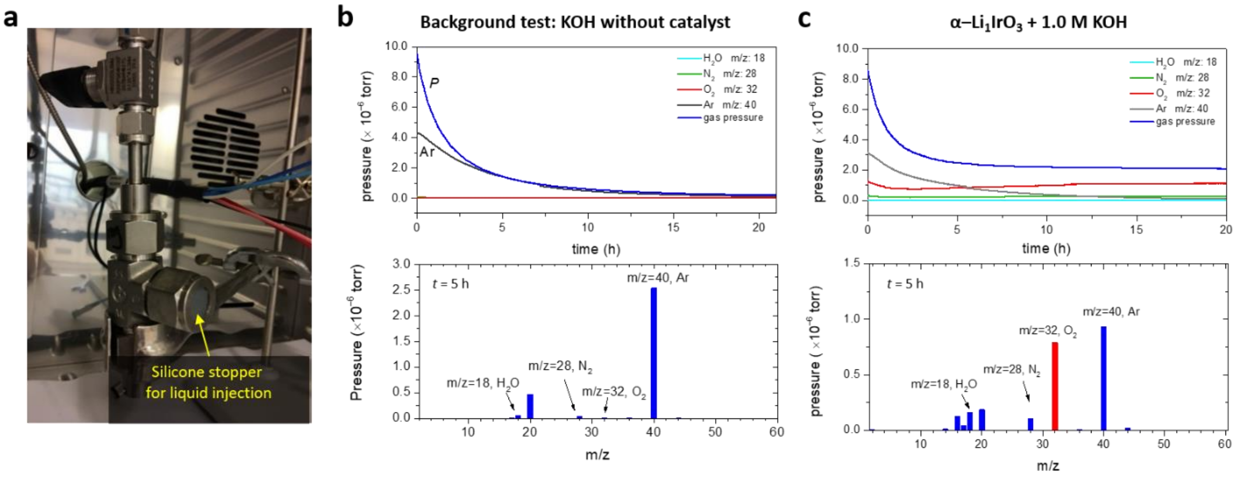

Supplementary Fig. 21. Online mass spectroscopy analysis of $O_{2(g)}$ generated during the chemical reaction of α-Li_1IrO_3 with KOH solution. The α-Li_1IrO_3 powder is prepared by oxidizing α-Li_2IrO_3 in H_2O-free Li-ion battery up to ~4.0 V. The powder is then washed with DMC solvent and ~50 mg of the obtained α-Li_1IrO_3 powder was kept in a sealed three-neck Swagelok cell and filled with Ar gas (a). 2 mL of Ar-saturated aqueous alkaline solutions with different KOH or NaOH concentrations was then injected into the Swagelok cell through a silicon rubber stopper. The system was further kept for 24 hours and the gas evolution inside the system was monitored by an online mass spectrometer (c) Background test was performed in order to ensure that no oxygen was detected through leakage from the cell (b).

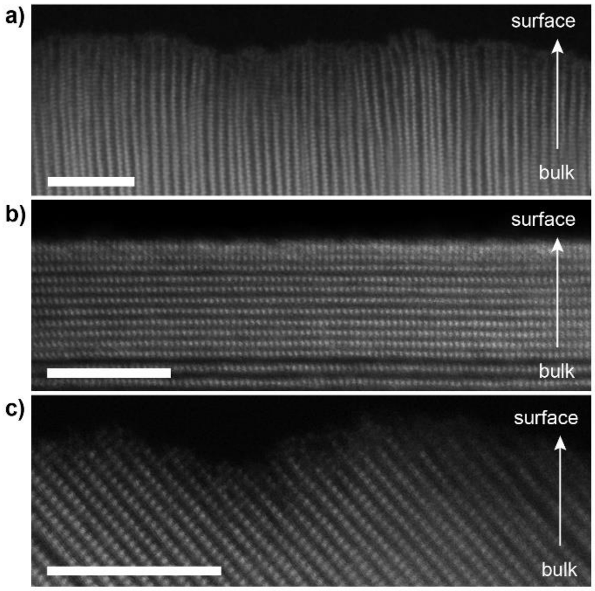

Supplementary Fig. 22. STEM images of the birnessite phase prepared by soaking α-Li_1IrO_3 in 1.0 M KOH overnight. (The scale bar is 5 nm.)

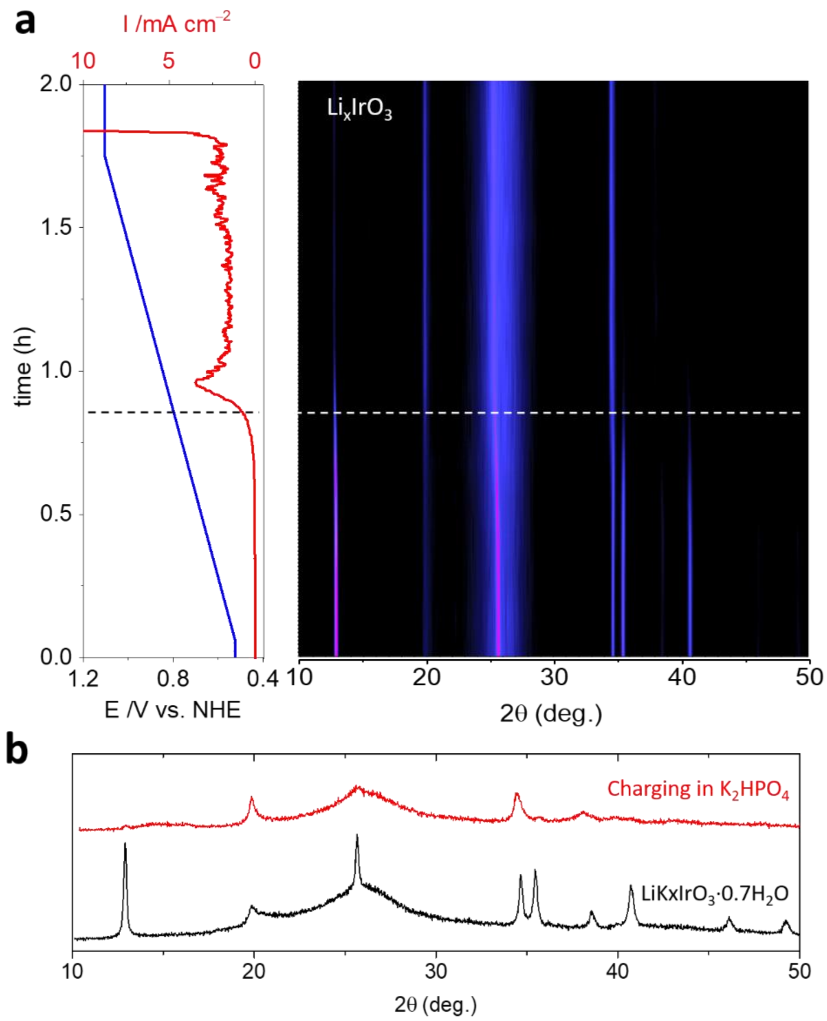

Supplementary Fig. 23. ***Operando*** **XRD measurement of the hydrated birnessite phase charging in an aqueous solution containing 0.25 M K_2HPO_4.**

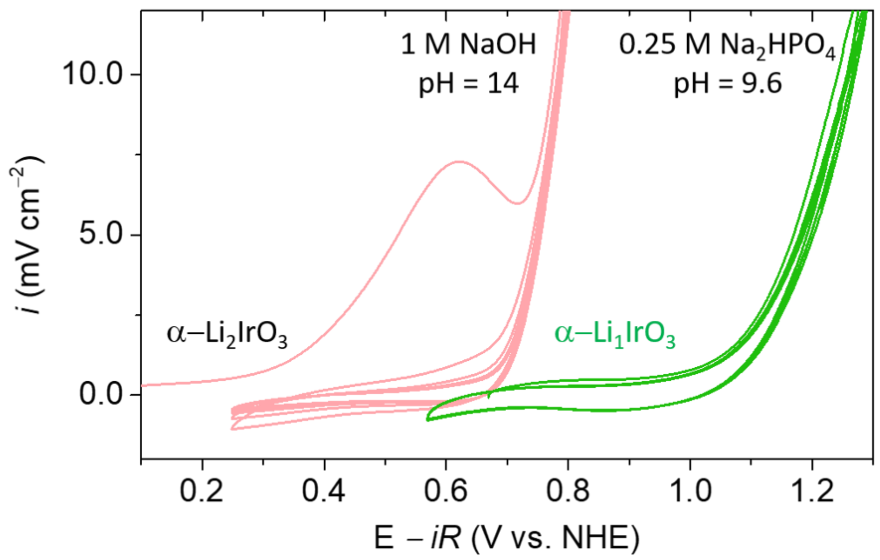

Supplementary Fig. 24. CV curves of α-Li_2IrO_3 first activated in 1.0 M NaOH solution (red curves) and then cycled in 0.25 M Na_2HPO_4 solution (green curves). No Na^+ intercalation into the structure of α-Li_2IrO_3 occurs during the electrochemical cycling in NaOH solution. Accordingly, there is no oxidation associated with cation de-intercalation in 0.25 M Na_2HPO_4 solution.

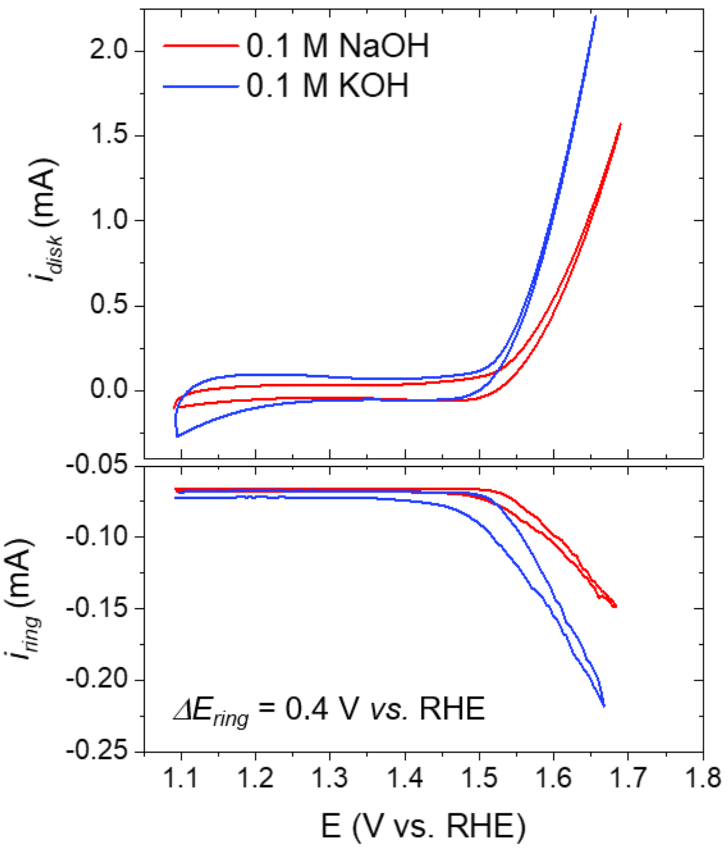

Supplementary Fig. 25. Cation effect and O_2 evolution efficiency studied by RRDE measurements. Cyclic voltammetry is conducted using α–Li_2IrO_3 as catalyst after activation for 10 cycles in 0.1 M KOH or NaOH solutions with stabilized current measured by the disk electrode.

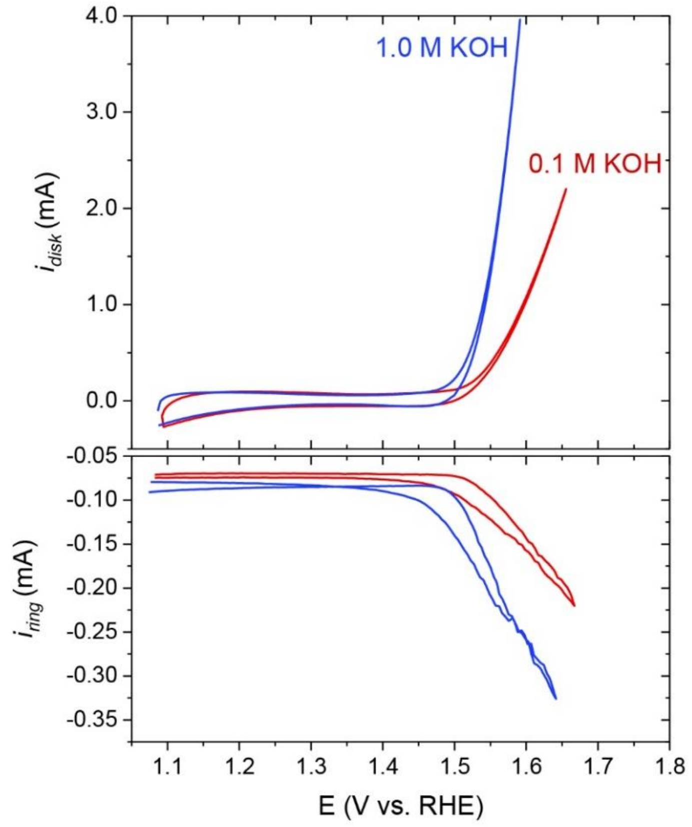

Supplementary Fig. 26. KOH concentration dependent OER activity and O_2 evolution efficiency studied by the RRDE measurement using the birnessite α-$LiK_{0.3}IrO_3·0.7H_2O$ as catalyst in Ar-saturated KOH solutions at different pH.

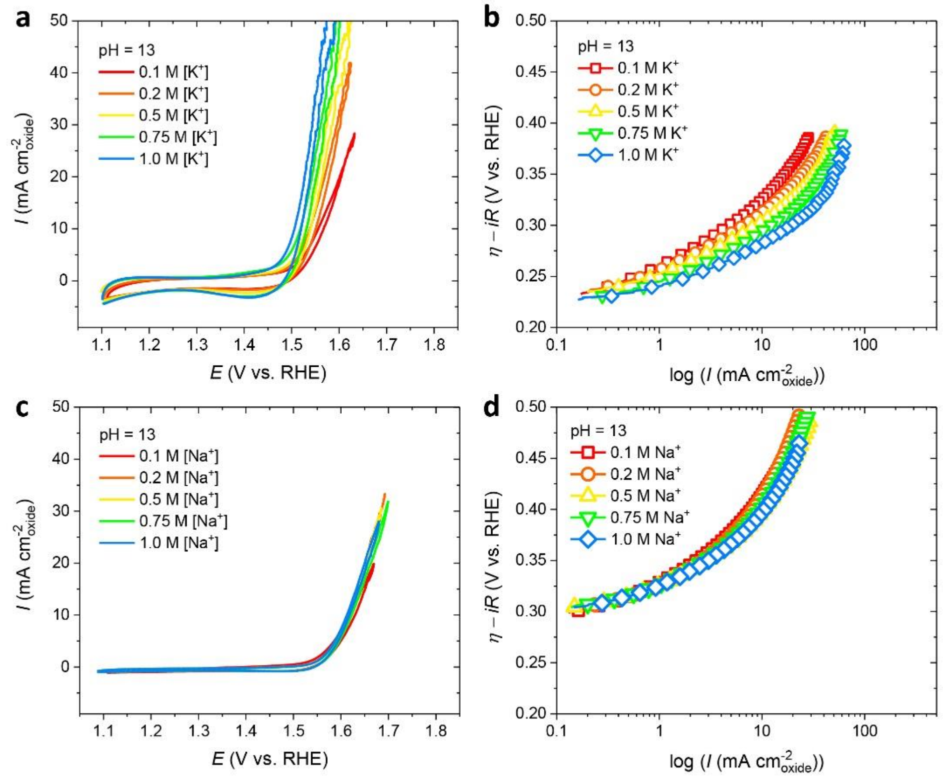

Supplementary Fig. 27. Correlation of the OER activities with [K^+] concentration. The concentration of [K^+] or [Na^+] in the testing electrolyte was gradually increased to 1.0 M by adding additional K_2SO_4 or Na_2SO_4 as supporting electrolytes while the pH of the solutions were maintained constant at 13.

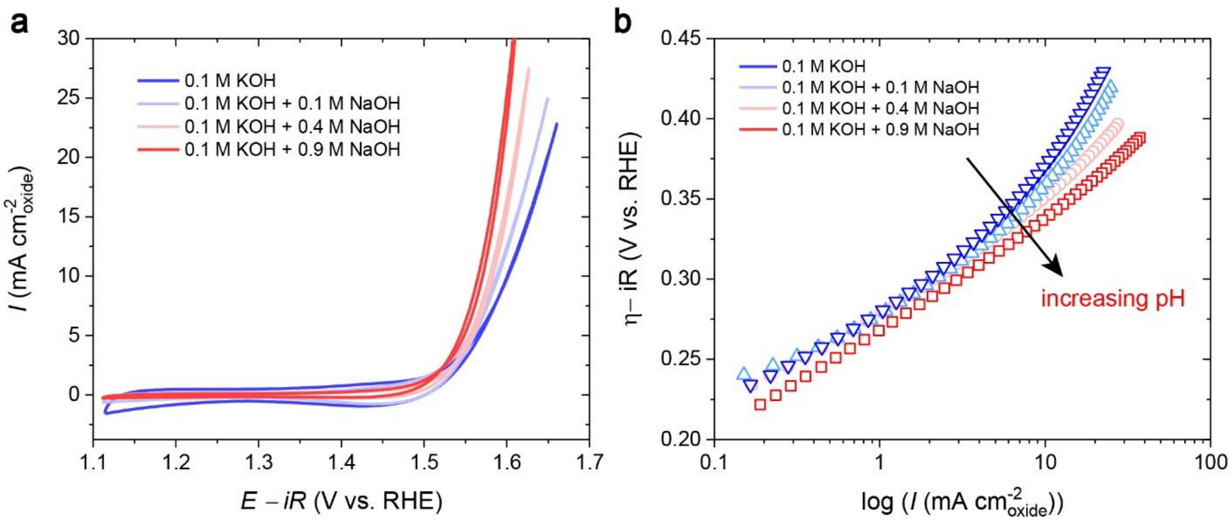

Supplementary Fig. 28. Correlation of the OER activities with [K^+] concentration and pH. The pH of the testing electrolyte was gradually adjusted to 14 by mixing NaOH and KOH while maintaining the concentration of [K^+] cations in the electrolyte constant to be 0.1 M. Alike [K^+], the [OH^-] concentration was also found to affect the OER kinetics. Nevertheless, it mostly affects the mass transport limited region at large overpotential.

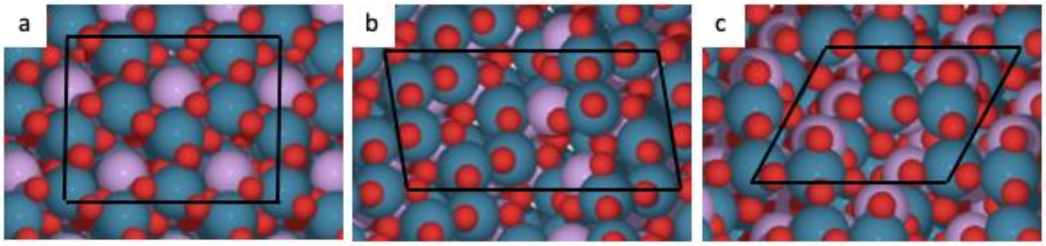

Supplementary Fig. 29. Surface models considered in this work, with the computational unit cells marked with black lines. a) 001 surface, b) armchair step (A-step) and c) zigzag-step (Z-step). Oxygen atoms are red, Li atoms pink and Ir atoms bluish-grey.

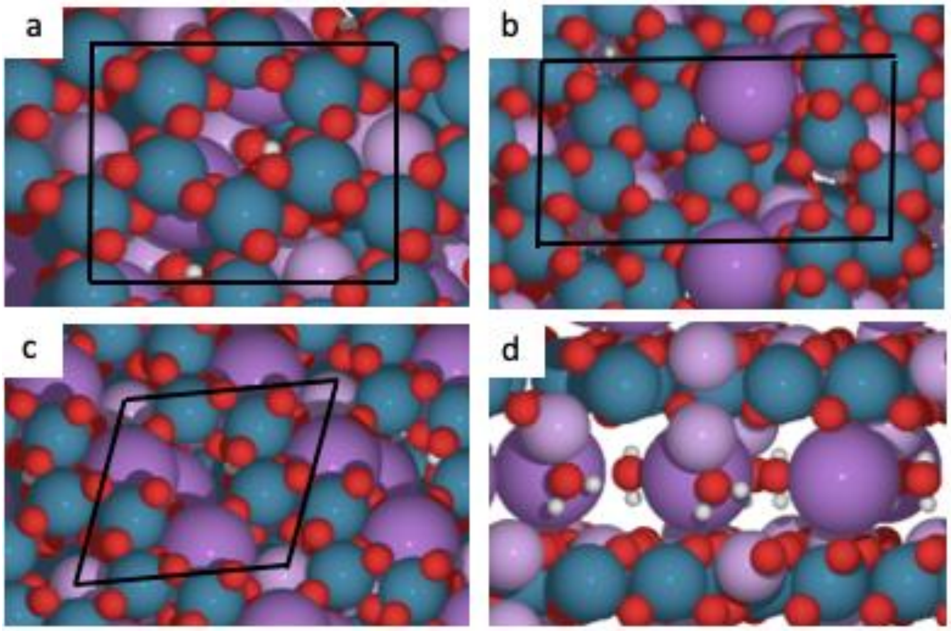

Supplementary Fig. 30: Surface structures for the disordered structures; a) 001 surface, b) A-step and c) Z-step. Li is pink, K is purple, O is red, Ir is bluish grey and H is white.

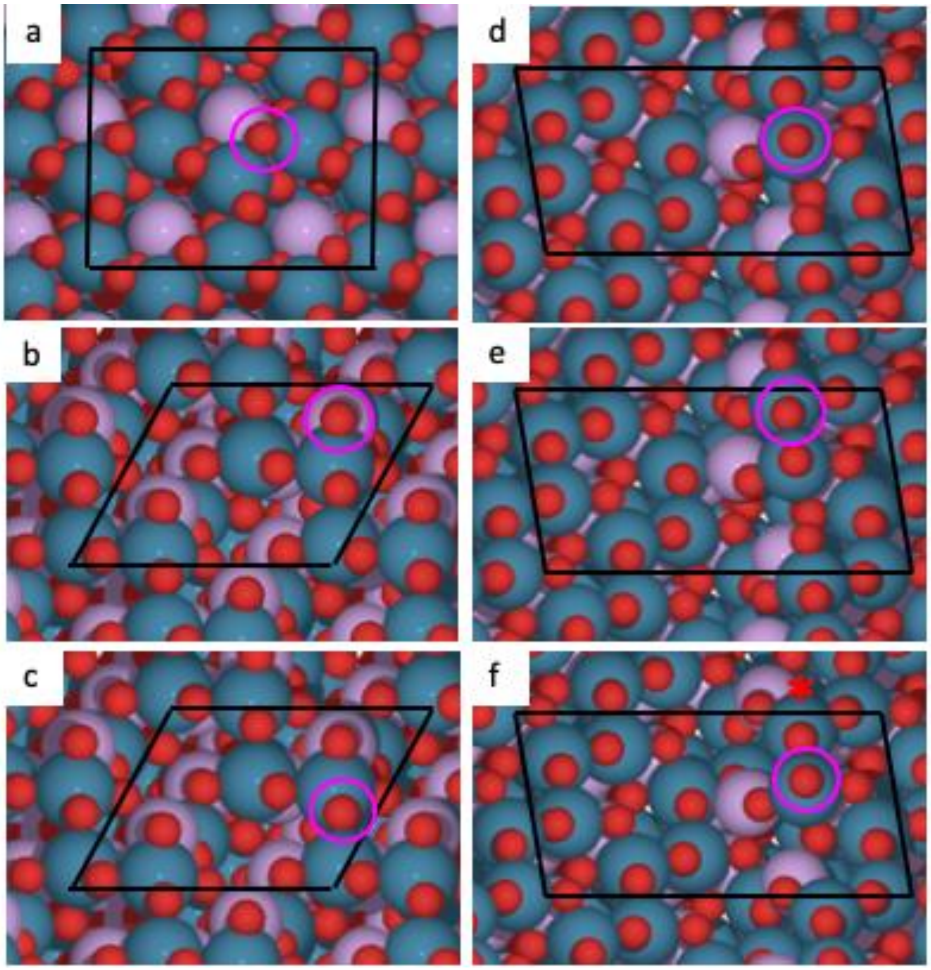

Supplementary Fig. 31. Sites considered as OER active sites. Magenta circles marks the O_{vac} site in the initial state. a) 001 surface site, b) Z-step position 1, c) Z-step position 2, d) A-step position 1, e) A-step position 2 and f) A-step with an Oxygen vacancy (marked by a red star).

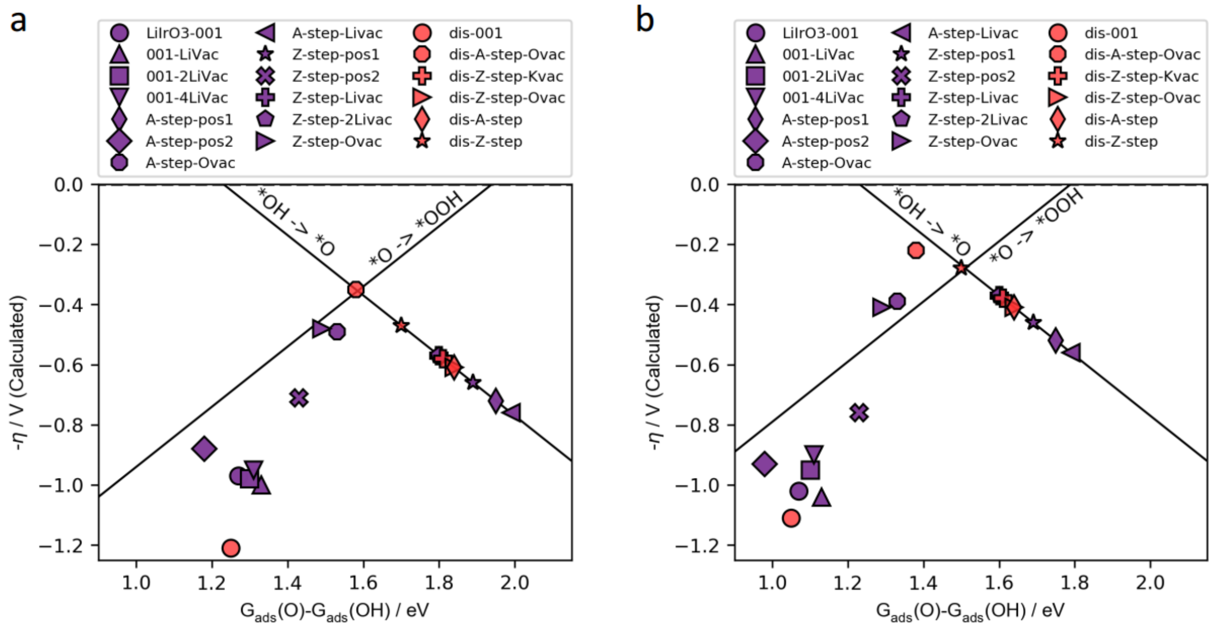

Supplementary Fig. 32. Volcano plot showing the limiting potential for various sites on the surface a) without and b) with estimated corrections for ZPE, entropy and solvation. Purple points correspond to sites on α-$LiIrO_3$ surfaces, some of which are illustrated in Figure S30, and red points are sites on the intercalated surface. Black lines indicate the scaling relations with the left leg drawn based on the average value of G_{ads}(OOH)-G_{ads}(OH) for all the calculated sites.

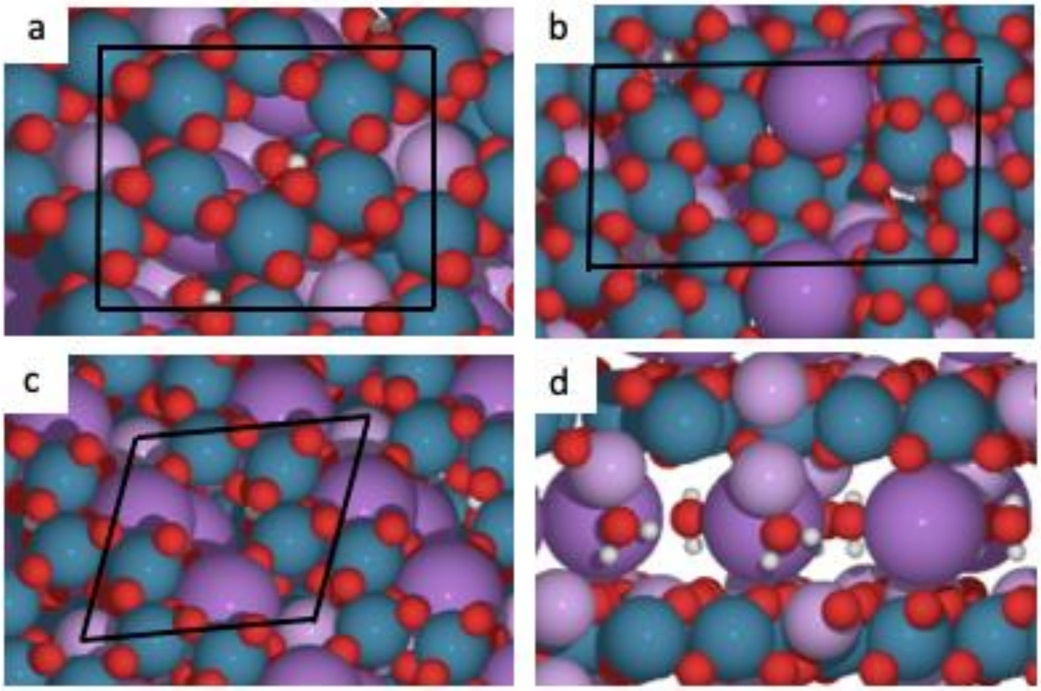

Supplementary Fig. 33. Surface structures for the disordered structures; a) 001 surface, b) A-step and c) Z-step. Li is pink, K is purple, O is red, Ir is bluish grey and H is white.

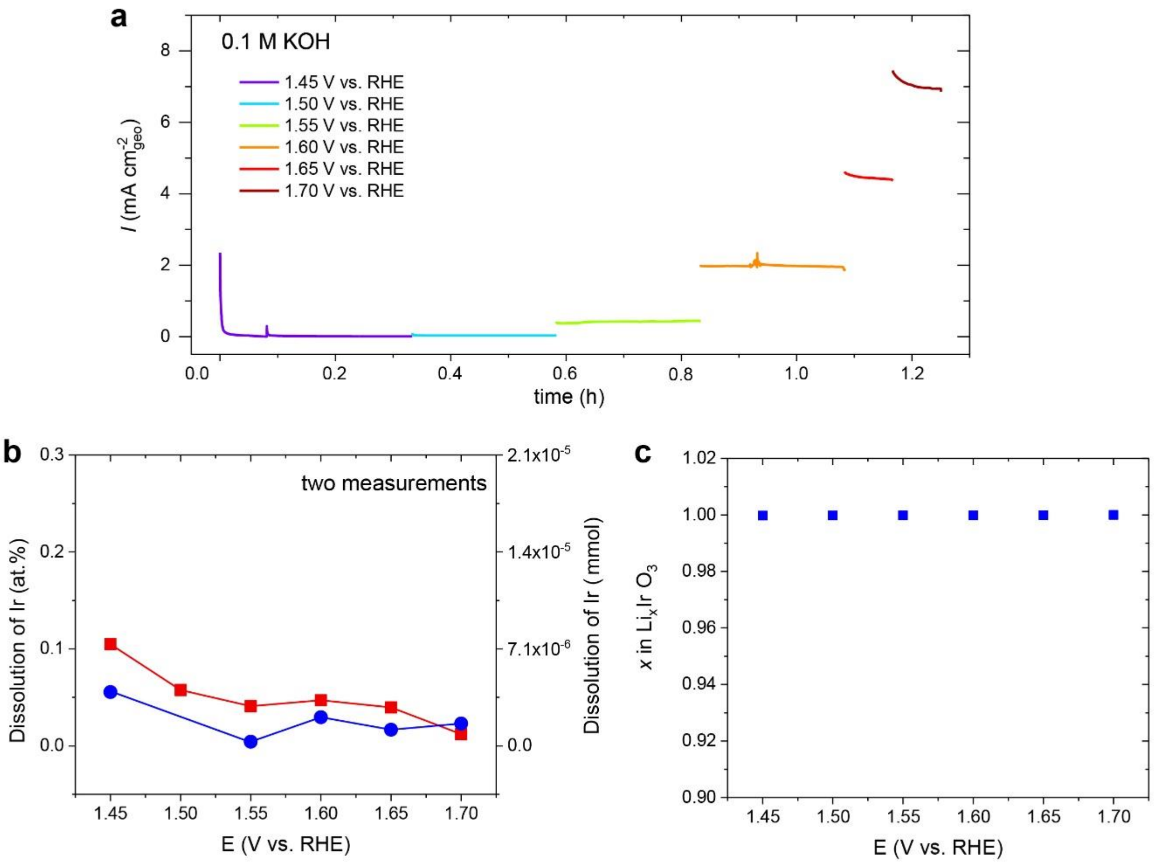

Supplementary Fig. 34. Ir and Li dissolution by ICP-OES measurement. Chronoamperometry was conducted by holding the working electrode at different potentials varying from 1.45 V to 1.70 V vs. RHE with each step for 20-40 mins. Note that the detection limit for Ir by the ICP-OES techniques is in the order of dozens of ppb, hence the results for Ir dissolution are within the detection limit of the machine and show that no, or almost no, iridium is dissolved upon OER.

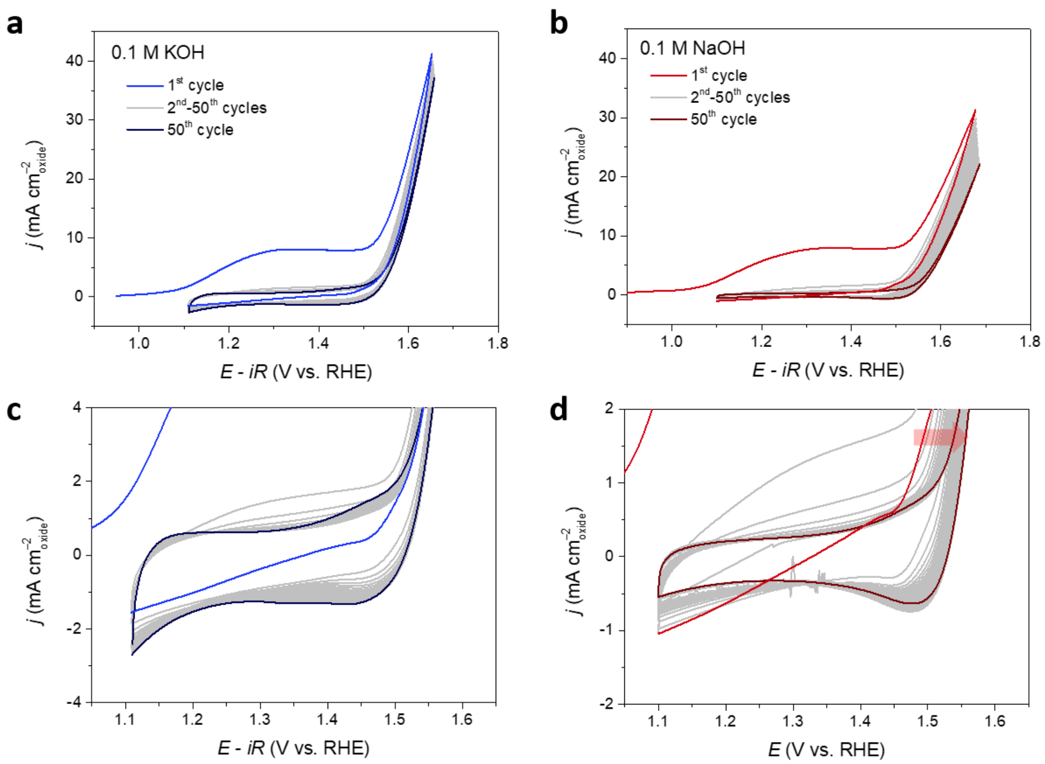

Supplementary Fig. 35. Electrochemical stability of α-Li_2IrO_3 cycled in (a,c) 0.1 M KOH and (b, d) NaOH for 50 cycles. As revealed by the XRD analyses, 1 equivalent of Li is delithiated from the structure during the initial CV cycle in both KOH and NaOH solutions to electrochemically activate the phase. A new phase is then formed in KOH solution while no Na^+-intercalation occurs in NaOH.

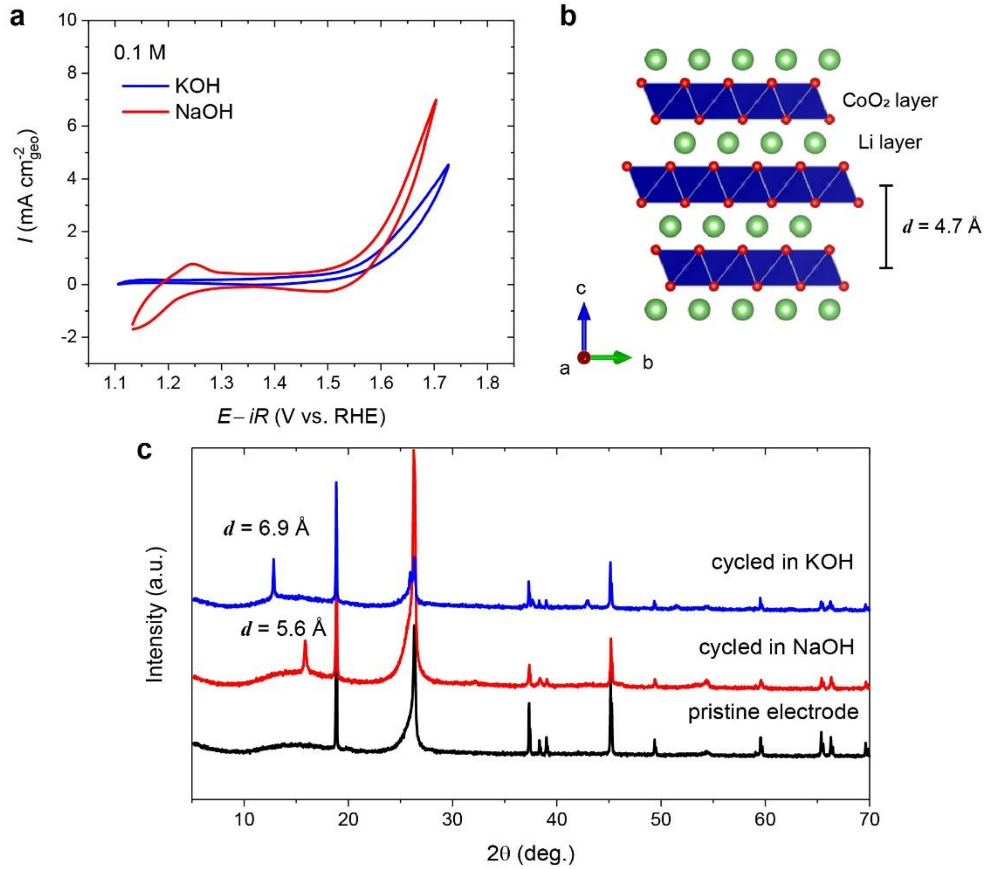

Supplementary Fig. 36. (a) Cyclic voltammetry, (b) crystallographic structure for the pristine $LiCoO_2$ and (c) XRD taken after OER measurements in 0.1 M KOH (blue curves) and 0.1 M NaOH (red curves) showing the modification of the interlayer distance, related to the intercalation of K^+ and Na^+ upon OER. The structure of $LiCoO_2$ is found to evolved upon cycling in KOH and NaOH with two different new phases forming, as indicated by the modification of the (003) peak at low angle. This result indicates that both K^+ and Na^+ intercalates into $LiCoO_2$ upon OER, leading to a cation dependent OER. These results demonstrate the universality of the EC mechanism described for α-Li_2IrO_3 that can be applied to other transition metal oxides.

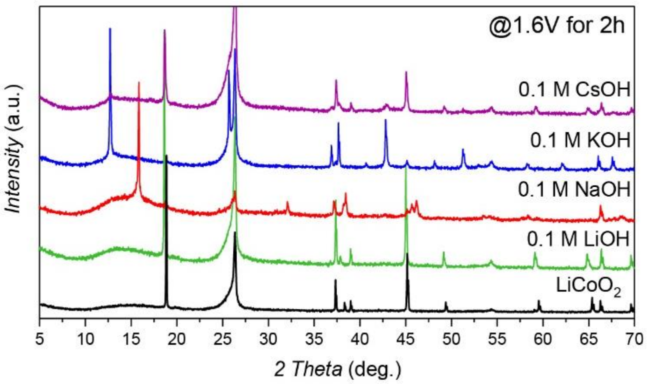

Supplementary Fig. 37. XRD profiles obtained for $LiCoO_2$ after being held at a constant voltage of 1.6 V vs. RHE in various 0.1 M alkaline solutions for 2 hours. Both Na^+ and K^+ cations can be selectively intercalated into the bulk layered structure, whereas Li^+ or Cs^+ cations cannot.

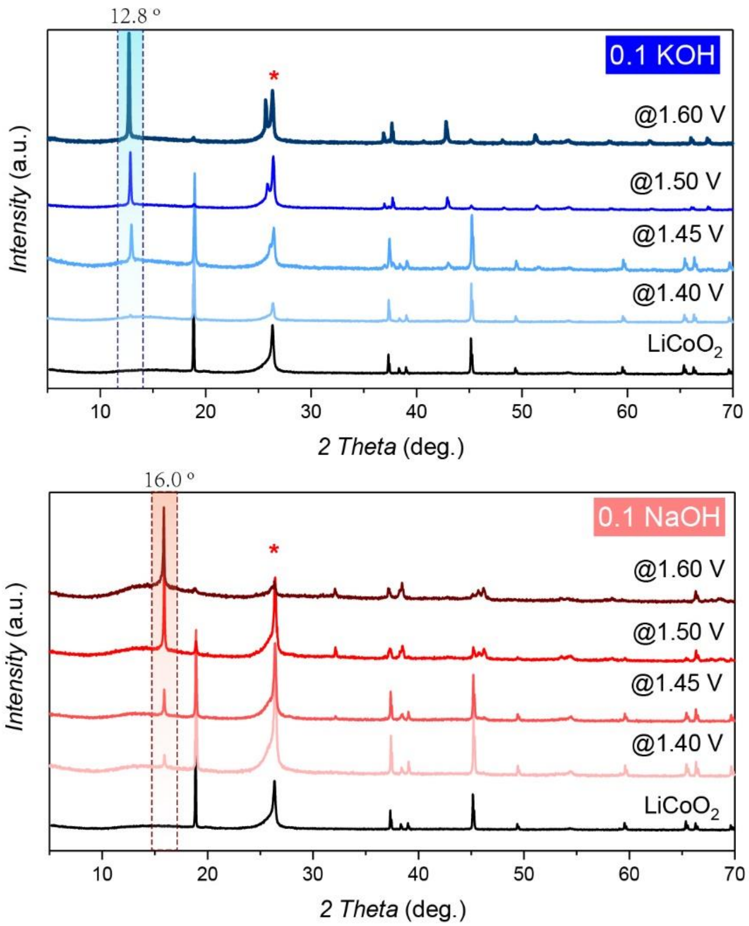

Supplementary Fig. 38. Structural evolution of $LiCoO_2$ in KOH and NaOH at different potentials. Na^+ and K^+ can be selectively intercalated into the bulk layered structure starting at 1.40 V and 1.45 V, respectively.

Supplementary Table 1. Surface area for the different Ir oxide catalysts as estimated by Brunauer-Emmett-Teller (BET) analyses.

Samples	Specific surface area (m^2 g^{-1})
α–Li_2IrO_3	0.10
β–Li_2IrO_3	0.085
$SrIrO_3$	0.42
IrO_2	0.26

Supplementary Table 2. Structural parameters for the birnessite O3-phase as deduced from the Rietveld refinement of laboratory and synchrotron XRD.

$Li_{0.5}K_{0.8}IrO_3{\cdot}0.7H_2O$<br>space group: *R*-3*m*<br>*a* = 3.03750(9) Å, *c* = 20.8279(14) Å<br>*V* = 166.42(2) Å^3, *Z* = 2					
atom	site	*x*	*y*	*z*	occ.
O1	6*c*	0	0	0.3909(7)	1
Ir/Li	3*a*	0	0	0	0.6667/0.3333
K/Ow	9*d*	0.5	0	0.5	0.533/0.466

Supplementary Table 3. Comparison of the calculated and experimentally determined unit cell parameters.

	Exp. (this work)	DFT (PBE)
a/Å	5.278	5.363 (+1,6%)
b/Å	8.974	9.029 (+0,6%)
c/Å	5.268	5.356 (+1,7%)
Beta/°	115.24	115.8

Supplementary Table 4. Li vacancy formation energy for the various surfaces assuming 1M Li in solution. The energies are per Li atom and differential energies, i.e. the energy required to make the second vacancy when the first vacancy has already been made, are given in parenthesis. Additional vacancies have only been considered if the differential vacancy formation energy is <0.30 eV.

eV/vacancy	Layer termination<br>[001]	Armchair step<br>[102]	Zigzag Step<br>[111]
1 Livac	0.18	0.30	0.13
2 Livac	0.25 (0.32)	x	0.21 (0.29)
3 Livac	x	x	0.34 (0.60)

Supplementary Table 5. Oxygen vacancy formation energies of the various surfaces relative to H_2O at U=0V. Corrections for ZPE and entropy are included.

eV/vacancy	[001]	A-step, pos. 1	A-step, pos. 2	Z-step, pos. 1	Z-step, pos. 2
O_{vac}	-0.86	-2.75	-1.79	-2.77	-1.45

Supplementary Table 6. Vacancy formation energies for the various surfaces of the disordered structure. The O_{vac} formation energies are relative to H_2O and include the corrections for ZPE and entropy.

eV/vacancy	[001]	A-step	Z-step
K vac (1M K^+)	x	0.74	0.85
O vac (U=0V)	-0.40	-2.51	-2.37

Supplementary Table 7: Calculated free energies for the reactions (1)-(4). All values are in eV. The limiting potential for each site is marked in red.

	ΔG_1	ΔG_2	ΔG_3	ΔG_4
$LiIrO_3$ [001]	-0.22	1.07	2.25	1.82
$LiIrO_3$ [001], 1 Li vac	-0.62	1.13	2.27	2.13
$LiIrO_3$ [001], 2 Li vac	-0.48	1.10	2.18	2.11
$LiIrO_3$ [001], 4 Li vac	-0.39	1.11	2.13	2.08
$LiIrO_3$ A-step, pos 1	1.01	1.75	1.04	1.12
$LiIrO_3$ A-step, pos 2	0.81	0.98	2.16	0.97
$LiIrO_3$ A-step, O vac	0.41	1.33	1.55	1.62
$LiIrO_3$ A-step, Li vac	0.97	1.79	1.03	1.13
$LiIrO_3$ Z-step, pos 1	1.08	1.69	1.25	0.90
$LiIrO_3$ Z-step, pos 2	0.22	1.23	1.99	1.48
$LiIrO_3$ Z-step, O vac	0.38	1.29	1.64	1.61
$LiIrO_3$ Z-step, Li vac	0.75	1.60	1.48	1.10
$LiIrO_3$ Z-step, 2 Li vac	0.81	1.60	1.42	1.10
Disordered, [001]	-0.65	1.05	2.18	2.34
Disordered, A-step O vac	0.66	1.38	1.45	1.43
Disordered, Z-step O vac	0.56	1.64	1.33	1.39
Disordered, Z-step K vac	1.16	1.61	1.39	0.76
Disordered, A-step	0.87	1.64	1.17	1.24
Disordered, Z-step	0.86	1.50	1.05	1.51

Supplementary Notes

Supplementary Note 1.

As shown in Supplementary Fig. 2, the α-Li_1IrO_3 and β-Li_1IrO_3 can be formed at a voltage of ~ 4.05 and 4.20 V vs. Li^+/Li, respectively, during the galvanostatic charging process in Li-ion battery after x = 1 lithium removal from the structure. These voltages are above the voltage of E = 3.503 V vs. Li^+/Li (as indicated in the above figure) which corresponds to the OER reversible potential at E = 1.23 V vs. RHE in alkaline solutions at pH = 13.

Supplementary Note 2.

As observed in Supplementary Fig. 3 When using α-Li_2IrO_3 as OER electrocatalyst in alkaline media (KOH or NaOH solutions), an oxidation event corresponding to the delithiation occurs in the potential range of ~1.0 – 1.5 V vs. RHE with approximately x =1.0 equivalent of lithium being removed from the structure. The formation of α-Li_1IrO_3 and β-Li_1IrO_3 after this oxidation event is then confirmed by ex situ XRD (Supplementary Fig. 7-8).

Interestingly, the electrochemically formed α-Li_1IrO_3 demonstrates different capacitive behavior in KOH than in NaOH. The capacitive envelope is found almost constant in the potential range 13-1.4 V, indicative that the electrochemically active surface area is not drastically modified by changing the supporting electrolyte and therefore cannot explain the enlarged OER activity measured with KOH (around 5 times greater at fixed current density) compared to NaOH. Only the pseudocapacitive behavior observed by the peaks below 1.25 V and above 1.4 V and presumably associated with cation adsorption/intercalation for α-Li_1IrO_3 in KOH increases by a factor of 1.5-2 for η comprised between 0.25 and 0.3 V but cannot account for the modification of the OER by a factor of ≈ 5.

Supplementary Note 3.

As observed in Supplementary Fig. 9, Starting from α-Li_2IrO_3, a first oxidation event occurs to form α-Li_1IrO_3 (blue pattern). Then, when the electrode potential is held at 1.55 V vs. RHE for 10 hours, a new phase starts to form (red pattern) with a characteristic peak appearing at 2θ = 12.6 deg., suggesting an expansion of the layered space from d = 4.7 Å to ≈ 6.9 Å during the OER.

Supplementary Note 4.

As observed in Supplementary Fig. 10, XRD measurements reveal that the hydrated birnessite phase α-$Li_1K_xIrO_3 \cdot 0.7H_2O$ formed by electrochemical cycling of α–Li_2IrO_3 catalyst in KOH solution as seen in Supplementary Fig. 8 can also be prepared in a chemical way. By soaking the α–Li_1IrO_3 powder in different alkaline solutions of 1.0 M KOH, NaOH, and LiOH, only the KOH solution was found to trigger the chemical reaction leading to the structural modification and the formation of the hydrated birnessite phase.

Supplementary Note 5

As observed in Supplementary Fig. 15, the mass loss starting below 100°C for the birnessite α-$LiK_{0.3}IrO_3·0.7H_2O$ is associated with the loss of structural water. Knowing the potassium amount to be 0.3 per iridium atom and assuming x to be equal to 1 for lithium, the loss of 4.2 w% corresponds to approximatively 0.7 H_2O per formual unit. The loss at around 450°C for the birnessite and 550°C for α-Li_1IrO_3 corresponds to the decomposition of the phase and the formation of IrO_2.

Supplementary Note 6

^{1}H solid-state NMR (ssNMR) analysis of the hydrated birnessite phase in Supplementary Fig. 16 shows that 89% of the protons detected is coming from water, and only 11% of proton is coming from OH^-. Based on the TGA analysis (Figure S18) and Rietveld analysis of the synchrotron XRD (Supplementary Table 2), the total amount of structural water per formula unit is 0.7. Considering that 11% of this structural water comes from OH^-, as seen by ssNMR, it would mean that 0.077 OH^- are inserted into the catalyst. This is not enough to counterbalance the 0.3 K^+ that are found to be intercalated. Hence, the charge neutrality is not kept and the phase is reduced upon chemical OER.

Supplementary Note 7

As observed in Supplementary Fig. 23, the birnessite phase was first prepared by soaking α-Li_1IrO_3 in KOH solution and then, the powder was recovered before to be assembled in the operando XRD cell with a 0.25 M K_2HPO_4 electrolyte. The potential was linearly scanned from OCV (~0.5 V) to 1.1 V vs. NHE at a scan rate of 0.1 mV s^{-1}. (b) selected XRD diffractograms of the as-prepared birnessite phase before (black curve) and after (red curve) charging in K_2HPO_4 solution. The characteristic peak at 2θ = 12.6° is found to be completely suppressed after the oxidation peak starting at around 0.7 V vs NHE, which is therefore associated with the de-intercalation of K^+ from the birnessite phase and the regeneration of the K^+-free catalyst.

Supplementary Note 8

As observed in Supplementary Fig. 25, RRDE measurements[15,16] were conducted to better understand the cation effect on the anodic current, and more specifically the current resulting from the oxygen evolution on the surface of the catalyst in different alkaline solutions. The RRDE electrode consists of a glassy carbon working electrode loaded with 50 μg $cm^{-2}{}_{geo}$ of α–Li_2IrO_3 catalyst and a Pt ring electrode. The collection efficiency is calculated as $\eta = \frac{i_{ring}}{i_{disk}} \times 100\%$. By calculating the collection efficiency $\eta = (i_{ring}/i_{disc})$ at a fixed potential (E = 1.58 V vs. RHE) at which the oxygen evolution occurs, the effective current contributing from oxygen evolution can thus be estimated.[16] While the theoretical collection efficiency would be 25 % based on the geometry of the RRDE electrodes, the use of drop-casted powder is known to lead to lower collection efficiency. Values ranging from 2 to 19% were previously reported in the literature for various OER catalysts.[15-19] As revealed by the RRDE analyses, the collection efficiency obtained in KOH and NaOH are 11.2% and 10.7%, respectively. As a result, we can conclude that the enhanced anodic current obtained in KOH is indeed related to a higher O_2 gas generation, suggesting an intrinsic high OER activity.

RRDE measurement was further carried out to better understand the effect of [KOH] on the anodic current and O_2 evolution efficiency. The collection efficiency obtained in 0.1 M and 1.0 M KOH are evaluated to be 11.2% and 9.6%, respectively, at E = 1.58 V vs. RHE. An intrinsic high OER activity can be obtained with high concentration of KOH when using the birnessite as catalyst.

Supplementary Note 9

As observed in Supplementary Fig. 27, the electrochemical behavior measured in NaOH and KOH are intrinsically different. Indeed, we further tested the effect of K^+ concentration in the electrolyte by adding K_2SO_4 salt and could confirm that the OER activity is dependent on the concentration of K^+, and that this concentration effect affects both the high overpotential mass transport limited region as well as the Tafel region at ower overpotential. Therefore, K^+ is involved in the rate determining step and is decoupled from the electron exchange. It is therefore in equilibrium between the structure and the solution and it is continuously shuttling (intercalate and deintercalate) on the solid catalyst/electrolyte interface between the bulk and the solution. No such effect was observed for Na^+, confirming that Na^+ is not involved into the OER reaction, unlike K^+.

Supplementary Note 10

For Supplementary Table 2, the relative occupancies were determined with the following procedure: i) there are 0.7 water molecules per Ir, ii) the potassium content (which is the heaviest atom contributing therefore the most to the diffracted intensity) is freely refined, iii) the Li ratio is fixed by considering that the 9*d* positon should be at maximum occupied at 1/3, and that the resulting chemical formulae should make sense regarding the Ir oxidation state comprised between 4+ and 5+ as determined by XAS.

The chemical composition as determined by Rietveld analysis for the O3 phase shows greater potassium content than what was determined by chemical analysis (EDX). This is explained by the formation of the O1 domains during the washing step. Indeed, while the chemical analysis probe the whole sample particles and therefore gives an average of the O3 and O1 domains created during the washing step, the Rietveld analysis only take into account the O3 domains and therefore a greater potassium amount is found.

Nevertheless, as our operando XRD study reveals (Fig. 3F in the manuscript), no such O1 domains are found during the electrochemical formation of the birnessite phase. Therefore, care must be exercised when considering the exact chemical composition for the birnessite phase which is constantly evolving depending on the conditions and the characterizations performed.

Supplementary Discussion

1. Possible Li and O surface vacancies in Li_1IrO_3

As shown in Fig 6 of the manuscript several different surface terminations are observed experimentally. We therefore consider the three different surfaces shown in Supplementary Fig. 29. Given the strongly oxidising conditions we furthermore consider the number of Li atoms in the subsurface layer of the [001] surface and near the step edge of the stepped surfaces. The Li vacancy formation energies for the surfaces are given in Supplementary Table 4. The values are calculated relative to Li(s) and corrected for the free energy of the reaction Li(s) → Li^+(aq, 1M) (-3.04 eV). For comparison the Li vacancy formation energy in a 2x1x3 unit cell of bulk α-Li_1IrO_3 is 0.20eV. The concentration of Li^+ in solution is not known and therefore the exact amount of Li at the surface can not be determined, however based on the loading of the catalyst and the volume of electrolyte a concentration of Li^+ of 4µM can be estimated. The free energy of dissolving Li(s) will then be reduced by kTln(4e-6) = 0.32 eV at room temperature. This suggests that 1-2 additional Li atoms could be removed from all three surfaces. The [001] surface is different from the steps since the vacancies are in the subsurface layer, and for our unit cell the removal of 4 Li atoms could result in a collapse of the subsurface Li layer. We therefore investigated this situation, however the vacancy formation energy was found to be 0.49 eV/vacancy making such a structure unfavourable.

As we start from an O-rich surface we consider the possibility of removing some of these to form surface oxygen vacancies. The O_{vac} formation energies at U=0V with H_2O as reference are given in Supplementary Table 5. Given that the OER will run at potentials above 1.23 eV only position 1 at the A-step and position 1 at the Z-step are plausible surface structures.

2. Possible K and O surface vacancies in $Li_{0.75}K_{0.25}(H_2O)_{0.50}IrO_3$

For the intercalated structure we consider the formation energy of oxygen and potassium vacancies at the surface and step edges, however limiting the number of structures based on our findings for α-Li_1IrO_3. Thus, we do not consider oxygen vacancies in position 2, since they are less favourable than vacancies at position 1, or sub-surface vacancies of the [001] surface, since they were found to have limited effect on the OER overpotential.

The structure of the subsurface layer means that there are in principle many different O sites, however the position relative to the subsurface layer is assumed to have limited effect on the binding energy compared with the surrounding IrO_x environment, and we therefore consider one site only. Furthermore, we only consider K vacancies at the step edges since there are no Li atoms right at the edge. The calculated vacancy formation energies are given in Supplementary Table 6, assuming a concentration of 1M K^+ for the K vacancies, and a potential of U=0V for the O vacancies. The K_{vac} formation energies are much higher than the Li_{vac} formation energies of α-Li_1IrO_3, and since the experiment is performed in 0.1M KOH the concentration of K^+ is higher than the concentration of Li^+, altogether making the formation of K vacancies unlikely. The formation of oxygen surface vacancies is also less favourable than on α-Li_1IrO_3, meaning that it is less likely for OER to happen close to another O_{vac}.

3. Active sites and OER activities

Since we start from an O-terminated surface, the formation of an active site requires the initial removal of an oxygen atom (i.e. formation of a ‘surface oxygen vacancy’). While the O atoms at the surface of the [001]-terminated α-$LiIrO_3$ are equivalent, there are several inequivalent O atoms at the step edges. In addition, the configuration of oxygen atoms around the Ir atoms at the step edges could differ from the octahedral configuration found in the bulk IrO_3 layers. Some of the inequivalent step sites considered here are illustrated in Supplementary Fig. 31. Structures with Li vacancies are not shown as these are in the subsurface or at the edge where they are difficult to visualize.

The OER limiting potentials for the calculated sites are plotted as a function of ΔG_2 in Supplementary Fig. 32a. The figure illustrates that the [001] termination has poor activity, and this is largely independent of the number of Li atoms removed from the subsurface layer. The stepped surfaces show much better activities. Supplementary Figure 31b shows the same results as Supplementary Fig. 32a but with the estimated corrections for ZPE, entropy and solvation added. The corrections result in some change in the values of the overpotentials, but the three structures that results in the lowest overpotentials for α-Li_1IrO_3 remain the same. The best limiting potential of 1.60 V is found for the Z-step with an additional lithium vacancy. The formation energy for this vacancy is 0.13 eV in 1M Li^+ (c.f. Supplementary Table 4), making it a realistic surface structure at the experimental conditions. The second and third most active sites are the step sites with oxygen vacancies, which are also plausible according to the formation energies in Supplementary Table 5.

For the intercalated $Li_{0.75}K_{0.25}(H_2O)_{0.50}IrO_3$ we calculate the OER limiting potential for some of the most plausible surface structures, i.e. the Z-step and the A-step with and without oxygen vacancies and the [001] surface. We also consider the less plausible Z-step with a K atom removed from the step edge for comparison with the best performing structure of α-Li_1IrO_3, the Z-step with an Li vacancy. The results are plotted as red markers in Supplementary Fig. 32, showing that all the sites at the steps have a high activity, while the [001] surface has a poor activity, comparable to the [001] surface of α-Li_1IrO_3. There are however no clear trend in the change of the adsorption energies and overpotential when comparing similar sites on the α-Li_1IrO_3 and the disordered surfaces.

4. Table of calculated adsorption energies

ΔG_1-ΔG_4 for all the surface sites considered are given in Supplementary Table 7. The results include the corrections for ZPE, entropy and solvation, and the limiting step in the reaction is highlighted. It is found that most of the sites are limited by the 2nd or 3rd step which define the activity volcano. However, if the corrections for ZPE, entropy and solvation are not included the energy of the *OOH intermediate is decreased and several of the sites are then limited by the 4th reaction step. We also note that including the corrections decrease the energy difference $G_{ads}(OOH)$-$G_{ads}(OH)$, thus making it possible to achieve a better overpotential within the scaling relations.

Supplementary Methods

Quantification of K^+ intercalation. The atomic ratio of K and Ir elements in the birnessite phase was evaluated by EDX analysis performed on a SEM FEI Quanta FEG 250 microscope. The amount of K intercalated into the layered structure is calculated based on the following equation:

Supplementary equation 1: $n_K = n_{Ir} \cdot x_{K/Ir} = \frac{m_{\alpha-Li1IrO3}}{M_{\alpha-Li1IrO3}} \cdot x_{K/Ir}$

in which, n_K is the amount of K (mol), n_K is the amount of Ir (mol), $x_{K/Ir}$ is the atomic ratio between K and Ir determined by the SEM-EDX, $m_{\alpha-Li1IrO3}$ is the mass of the as-prepared α-Li_1IrO_3 powder, and $M_{\alpha-Li1IrO3}$ is the molar mass of α-Li_1IrO_3 (247.16 g/mol).

It is worth to stress out that the K amount is very sensitive to the experimental conditions for preparing the birnessite phase (α-$Li_1K_xIrO_3 \cdot 0.7H_2O$), since the K cations can be easily leaching out from the structure during the washing step by using DI water. Hence, in order to limit the K leaching, a mixed solution of acetone and water (v/v, 1:1) was used for the washing step.

Dertermination of O_2 gas evolution. O_2 gas evolution when soaking the as-prepared α-Li_1IrO_3 powder in KOH solution was analyzed by the use of online mass spectrometry. The evolved O_2 gas amount is estimated using ideal gas law:

Supplementary Equation 2: $n_{O2} = \frac{P_{O2} \cdot V_{cell}}{RT} = P_{cell} \frac{P_{O2}'}{P'} \cdot \frac{V_{cell}}{RT}$

with n_{O2} being the amount of O_2 gas evolved (mol), P_{O2} the partial pressure of the O_2 gas in the Swagelok-type cell. P_{O2}' is the partial pressure of the m/z = 32 signal and P' is the gas pressure measured after the capillary inlet by the instrument. P_{cell} is the pressure of the Swagelok-type cell monitored by an external pressure sensor. Doing so, we assume that $\frac{P_{O2}}{P_{cell}} = \frac{P_{O2}'}{P'}$.

Computational details

Further details on the setup of the surfaces. The three surfaces considered for α-Li_1IrO_3 are shown in Supplementary Fig. 29; a 1x2 model of the [001] surface which corresponds to a termination that follows the layers (Supplementary Fig. 29a), a [102] termination which results in an armchair-type edge of the hexagonal IrO_3 layer (A-step, Supplementary Fig. 29b), and a [111] termination which results in a zigzag-type edge of the IrO_3 layer (Z-step, Supplementary Fig. 29c). All surfaces are modelled by 3 layer slabs of the bulk structure. The bottom of the slab is terminated by half a layer of Li which, along with the bottom layer of IrO_3, is fixed in the bulk position to resemble the bulk of the material. The step sizes are chosen such that the unit cell dimensions in the xy-plane are ca. 10x10Å.

In order to compare the performance of α-$LiIrO_3$ with that of the disordered material obtained in KOH solution we make a model of the intercalated structure. This is not a straightforward procedure given the many degrees of freedom, e.g in the ordering of K^+, Li^+ and H_2O in the spacer layers between the IrO_3 layers and in the orientation of the water molecules. Several different optimisation attempts were therefore performed in order to arrive at a structure where no significant rearrangements in the spacer layers occurred when different adsorbates were added to the surface.

The surface structure is based on the experimentally determined crystal structure, but expanded in a (2√3x√3x1/3) supercell, such that each Ir layer contains 4 Ir atoms, 2 Li atoms and 12 O atoms. The reduction in the c lattice parameter to 1/3 is necessary in order to create a stepped surface, since the layers may otherwise be translated relative to each other during initial optimisation,

creating a mismatch at the periodic boundaries. In the spacer layers 2 K atoms, 2 Li atoms and 2 H_2O molecules are initially positioned in an ordered fashion, then half of the Li and K atoms are removed to create a structure with stoichiometry $Li_{0.75}K_{0.25}(H_2O)_{0.50}IrO_3$ corresponding reasonably well with the experimentally determined stoichiometry of $Li_xK_{0.3}(H_2O)_{0.70}IrO_3$ ($x \leq 1$) and having the same charge balance as Li_1IrO_3. This structure forms the basis for the surfaces, which are made to correspond with the surfaces of α-Li_1IrO_3, i.e. a [001] surface, a zigzag step and an armchair step. The optimised structures of the surfaces, shown in Supplementary Fig. 30a-c, are found to have layer spacings of ca. 7.2 Å in reasonable agreement with the experimentally determined layer spacing of 6.94 Å. The structure of the spacer layer in the optimised surfaces is highlighted in Supplementary Fig. 30d. The oxygen atoms of the water molecules are found close to the positively charged K and Li atoms, and the hydrogens point towards the oxygen atoms of the IrO_3 layers. We note that this structure might not be the global minimum, but an extended search covering all degrees of freedom is beyond the scope of this paper. To limit the openness of the stepped structure the steps are terminated by K atoms.

Supplementary References

1. Lee Y, Suntivich J, May KJ, Perry EE, Shao-Horn Y. Synthesis and Activities of Rutile IrO_2 and RuO_2 Nanoparticles for Oxygen Evolution in Acid and Alkaline Solutions. *J. Phys. Chem. Lett.* **3**, 399-404 (2012).

2. Suntivich J, May KJ, Gasteiger HA, Goodenough JB, Shao-Horn Y. A Perovskite Oxide Optimized for Oxygen Evolution Catalysis from Molecular Orbital Principles. *Science* **334**, 1383-1385 (2011).

3. Grimaud A, *et al.* Double perovskites as a family of highly active catalysts for oxygen evolution in alkaline solution. *Nat. Commun.* **4**, 2439 (2013).

4. Diaz-Morales O, *et al.* Iridium-based double perovskites for efficient water oxidation in acid media. *Nat. Commun.* **7**, 12363 (2016).

5. Geiger S, *et al.* The stability number as a metric for electrocatalyst stability benchmarking. *Nat. Catalysis*, (2018).

6. Yagi S, *et al.* Covalency-reinforced oxygen evolution reaction catalyst. *Nat. Commun.* **6**, 8249 (2015).

7. Seitz LC, *et al.* A highly active and stable $IrO_x/SrIrO_3$ catalyst for the oxygen evolution reaction. *Science* **353**, 1011-1014 (2016).

8. Grimaud A, *et al.* Activating lattice oxygen redox reactions in metal oxides to catalyse oxygen evolution. *Nat. Chem.* **9**, 457-465 (2017).

9. Kim H, *et al.* Coordination tuning of cobalt phosphates towards efficient water oxidation catalyst. *Nat. Commun.* **6**, 8253 (2015).

10. Mefford JT, *et al.* Water electrolysis on $La_{1-x}Sr_xCoO_{3-delta}$ perovskite electrocatalysts. *Nat. Commun.* **7**, 11053 (2016).

11. McCrory CCL, Jung S, Ferrer IM, Chatman SM, Peters JC, Jaramillo TF. Benchmarking Hydrogen Evolving Reaction and Oxygen Evolving Reaction Electrocatalysts for Solar Water Splitting Devices. *J. Am. Chem. Soc.* **137**, 4347-4357 (2015).

12. Stoerzinger KA, Qiao L, Biegalski MD, Shao-Horn Y. Orientation-Dependent Oxygen Evolution Activities of Rutile IrO_2 and RuO_2. *J. Phys. Chem. Lett.* **5**, 1636-1641 (2014).

13. Ng JWD, *et al.* Gold-supported cerium-doped NiO_x catalysts for water oxidation. *Nat. Energy* **1**, 16053 (2016).

14. McCrory CC, Jung S, Peters JC, Jaramillo TF. Benchmarking heterogeneous electrocatalysts for the oxygen evolution reaction. *J. Am. Chem. Soc.* **135**, 16977-16987 (2013).

15. Filimonenkov IS, Istomin SY, Antipov EV, Tsirlina GA, Savinova ER. Rotating ring-disk electrode as a quantitative tool for the investigation of the oxygen evolution reaction. *Electrochimi. Acta* **286**, 304-312 (2018).

16. Köhler L, Ebrahimizadeh Abrishami M, Roddatis V, Geppert J, Risch M. Mechanistic Parameters of Electrocatalytic Water Oxidation on $LiMn_2O_4$ in Comparison to Natural Photosynthesis. *ChemSusChem* **10**, 4479-4490 (2017).

17. Scholz J, Risch M, Stoerzinger KA, Wartner G, Shao-Horn Y, Jooss C. Rotating Ring–Disk Electrode Study of Oxygen Evolution at a Perovskite Surface: Correlating Activity to Manganese Concentration. *J. Phys. Chem. C* **120**, 27746-27756 (2016).

18. Vos JG, Koper MTM. Measurement of competition between oxygen evolution and chlorine evolution using rotating ring-disk electrode voltammetry. *J. Electroanal. Chem.* **819**, 260-268 (2018).

19. Yang C, *et al.* Revealing pH-Dependent Activities and Surface Instabilities for Ni-Based Electrocatalysts during the Oxygen Evolution Reaction. *ACS Energy Lett.* **3**, 2884-2890 (2018).
